# Local introduction and heterogeneous spatial spread of dengue-suppressing *Wolbachia* through an urban population of *Aedes aegypti*

**DOI:** 10.1371/journal.pbio.2001894

**Published:** 2017-05-30

**Authors:** Tom L. Schmidt, Nicholas H. Barton, Gordana Rašić, Andrew P. Turley, Brian L. Montgomery, Inaki Iturbe-Ormaetxe, Peter E. Cook, Peter A. Ryan, Scott A. Ritchie, Ary A. Hoffmann, Scott L. O’Neill, Michael Turelli

**Affiliations:** 1 School of BioSciences, Bio21 Institute, University of Melbourne, Parkville, Victoria, Australia; 2 Institute of Science and Technology, Klosterneuburg, Austria; 3 Institute of Vector-Borne Disease, Monash University, Clayton, Victoria, Australia; 4 School of Public Health, Tropical Medicine and Rehabilitation Sciences, James Cook University, Cairns, Queensland, Australia; 5 Department of Evolution and Ecology, University of California, Davis, Davis, California, United States of America; Pennsylvania State University, United States of America

## Abstract

Dengue-suppressing *Wolbachia* strains are promising tools for arbovirus control, particularly as they have the potential to self-spread following local introductions. To test this, we followed the frequency of the transinfected *Wolbachia* strain *w*Mel through *Ae*. *aegypti* in Cairns, Australia, following releases at 3 nonisolated locations within the city in early 2013. Spatial spread was analysed graphically using interpolation and by fitting a statistical model describing the position and width of the wave. For the larger 2 of the 3 releases (covering 0.97 km^2^ and 0.52 km^2^), we observed slow but steady spatial spread, at about 100–200 m per year, roughly consistent with theoretical predictions. In contrast, the smallest release (0.11 km^2^) produced erratic temporal and spatial dynamics, with little evidence of spread after 2 years. This is consistent with the prediction concerning fitness-decreasing *Wolbachia* transinfections that a minimum release area is needed to achieve stable local establishment and spread in continuous habitats. Our graphical and likelihood analyses produced broadly consistent estimates of wave speed and wave width. Spread at all sites was spatially heterogeneous, suggesting that environmental heterogeneity will affect large-scale *Wolbachia* transformations of urban mosquito populations. The persistence and spread of *Wolbachia* in release areas meeting minimum area requirements indicates the promise of successful large-scale population transformation.

## Author summary

*Wolbachia* are bacteria that live inside insect cells. In insects that act as viral vectors, *Wolbachia* can suppress virus transmission to new hosts. *Wolbachia* have been experimentally introduced into *Aedes aegypti* mosquito populations to reduce the transmission of dengue, Zika, and other arboviruses that cause human disease. *Wolbachia* invade populations by causing cytoplasmic incompatibility, a phenomenon whereby embryos from crosses between infected males and uninfected females fail to hatch. While *Wolbachia* have been shown to successfully invade and remain established in isolated *Ae*. *aegypti* populations, outward spread from urban release zones has not been previously documented. This is an important step in demonstrating that *Wolbachia* can be used to combat mosquito-borne infectious disease in cities. Here we describe *Wolbachia* spread from 2 introduction areas within Cairns in northeastern Australia at a rate of about 100–200 meters per year. Spread occurs only when introduction areas are sufficiently large. The slow rates of observed spread are broadly consistent with mathematical predictions based on estimated *Ae*. *aegypti* dispersal distances, *Wolbachia* dynamics, and effects seen in isolated populations. Spread is uneven and likely depends on local characteristics (e.g., barriers) that affect mosquito density and dispersal. Our data indicate that *Wolbachia* can be introduced locally in large cities, remain established where released, and slowly spread from release areas. These dynamics indicate that high *Wolbachia* infection frequencies can be established gradually across large urban areas through local releases.

## Introduction

Dengue fever is the most common arboviral disease affecting humans [1]. Over 2,500,000,000 people live in dengue-afflicted regions, and dengue incidence is increasing at an alarming rate in tropical and subtropical countries [2]. A number of other arboviruses also represent emerging disease risks, including chikungunya and Zika, the latter being associated with a recent explosive epidemic in South America [3,4]. The main approach to controlling these diseases has been suppression of the principal mosquito vector, *Ae*. *aegypti*, either through source reduction or insecticide-based control programs. Given the increasing incidence of *Ae*. *aegypti*-associated human disease, it is clear that current control measures are insufficient. In response to this problem, a number of new control approaches are currently being developed and tested [5,6,7,8,9,10].

In contrast to control efforts that require repeated population suppression, the Eliminate Dengue Program (http://www.eliminatedengue.com/program) aims to modify populations using long-lasting local introductions of a dengue-inhibiting *Wolbachia* into naturally uninfected populations of *Ae*. *aegypti*. The strain, *w*Mel, was transferred from *Drosophila melanogaster* into laboratory-raised *Ae*. *aegypti*, who inherit the infection maternally [11,12]. Following introgression of the infection into a native genetic background, *Wolbachia*-infected mosquitoes are released into the field to mate with wild uninfected mosquitoes, and *w*Mel frequency increases through cytoplasmic incompatibility (CI) [13,14]. CI describes the fact that uninfected females mated with *Wolbachia*-infected males produce inviable embryos. In *Ae*. *aegypti*, this is believed to occur in 100% of these incompatible crosses [11]. In contrast, infected females can mate with either infected or uninfected males and produce almost 100% infected progeny. CI greatly reduces the relative fitness of uninfected females when infected males are common and drives rapid establishment of *Wolbachia* in isolated mosquito populations [14], given that there is no mating bias against *w*Mel-infected *Ae*. *aegypti* [15].

Although *w*Mel-infected females receive a frequency-dependent relative fitness advantage from CI, they also suffer from frequency-independent fitness costs, including decreases in fecundity and larval competitive ability [16,17,18,19]. Thus, CI does not produce a net fitness advantage while *w*Mel is rare, resulting in dynamics analogous to those produced by an Allee effect in ecology [20,21] and by natural selection on a locus (or alternative karyotypes) in which heterozygotes are less fit than either homozygotes (i.e., underdominance, [22,23,24]). The interaction of the frequency-dependent advantage associated with CI and the frequency-independent cost(s) produces “bistable dynamics” with a threshold frequency of infection (denoted $\hat{p}$) below which the infection will be locally eliminated and above which frequencies systematically increase [25,26,27].

Curtis [23] first proposed transforming pest populations by introducing translocations that are expected to show bistable dynamics (cf. [28]). The bistable model for *Wolbachia* spread was introduced by Turelli and Hoffmann [29] to explain the rapid spread of *w*Ri, a CI-causing *Wolbachia* variant, through California populations of *Drosophila simulans*. Although this interpretation of *w*Ri dynamics has now been challenged by more recent data on the spread of natural *Wolbachia* infections [30], 3 lines of evidence nevertheless support bistability of *w*Mel-transinfected *Ae*. *aegypti* [31]: (1) frequency dynamics from the original field releases [14], (2) direct experimental evidence for lower fecundity and viability [19,32], and (3) new data showing that persistent influx over 2 years of *w*Mel-infected *Ae*. *aegypti* into a relatively isolated population has not led to establishment of *w*Mel there [31].

In order for the invasion to spread spatially under bistability, new uncolonised areas must receive infected immigrants at a rate high enough to be pushed past the threshold frequency, $\hat{p}$. Under the dynamics produced by CI-inducing *Wolbachia*, spatial spread is expected in a habitat with relatively homogeneous population densities if $\hat{p}$ is below a critical value near 0.5 [20,29]. For *w*Mel in *Ae*. *aegypti* near Cairns, $\hat{p}$ is thought to be moderate ($\hat{p}\approx 0.2–0.35$) because of its relatively low fitness costs and near-perfect maternal transmission [11,14,31].

Previously, *w*Mel-infected *Ae*. *aegypti* released in 2 relatively isolated communities in Northern Queensland, Australia (Gordonvale and Yorkeys Knob), colonised each area rapidly [14], and the infection has persisted at high frequency (>90%) at both sites [18]. Moreover, *w*Mel continues to show strong blockage of dengue transmission in laboratory-challenged mosquitoes derived from field collections [33]. Here we present data from 3 subsequent releases of *w*Mel-infected *Ae*. *aegypti* in Cairns, Northern Queensland, a city with about 150,000 residents that is located between the communities of Gordonvale and Yorkeys Knob. These releases followed protocols similar to those of [14], but the release zones were centred within suburban landscapes, providing a continuous habitat for *Ae*. *aegypti*. This study investigates the capability of the *w*Mel infection to spread spatially through urban *Ae*. *aegypti* populations and the stability of the infection in invaded regions over time.

Spread from localized releases to surrounding uninfected areas depends on mosquito dispersal and relative population densities. Spatial spread can be slowed or stopped if densities are higher in surrounding uninfected areas [20]. Dispersal of *Ae*. *aegypti* varies with local environmental conditions. Poor habitats generally induce larger dispersal distances as gravid females must travel further to find the relatively rare oviposition sites [34,35,36]. Despite its global success as an invasive species in tropical habitats, presumably through dispersal of eggs and larvae [37], adult *Ae*. *aegypti* are generally considered weak dispersers. Females usually remain within 50–150 m of their eclosion site [34,38,39,40,41,42]. They appear to disperse poorly across highways [31,42,43] and through vegetated parkland [44]. Occasional long-range dispersal, on the order of 0.5–1 km, has been observed [45,46,47,48]. However, given the bistable dynamics of *w*Mel in *Ae*. *aegypti*, rare long-range dispersal will not accelerate *Wolbachia* spread because the infection will not increase locally from low initial frequencies [20,31].

We document local *w*Mel establishment and heterogeneous spatial spread from the 2 relatively large release areas. Our new data demonstrate that local *Wolbachia* introductions can succeed, persist for at least 2 years, and produce slow spatial spread. Using graphical summaries, we approximate the rate of spatial spread and the width of the spreading wave. We also show that our field data are broadly consistent with simple mathematical models that depend critically on bistable frequency dynamics for *w*Mel transinfected into *Ae*. *aegypti*. These models involve only 2 parameters, one describing the position of the unstable threshold point, $\hat{p}$, and the other, *σ*, describing average *Ae*. *aegypti* dispersal distance. Both parameters can be estimated independently of spread data [31]. We also present likelihood-based data analyses that fit simple curves to estimate the shape and speed of *Wolbachia* spread. The shape of the advancing wave is summarized by wave width, defined as the inverse of the maximum slope in infection frequencies, averaged over the wave front [49]. As discussed below, wave width provides an estimate of dispersal distance averaged over time. Wave speed is defined as the average rate of movement of an intermediate infection frequency (e.g., 0.5.) The theory of bistable waves leads to a simple prediction for wave speed in terms of wave width and $\hat{p}$, the threshold infection frequency above which local increases in infection frequencies (*p*) are expected [20,24,31,50]. The observed speed of *w*Mel spread in Cairns is broadly compatible with this prediction, and the estimated wave width is also consistent with independent estimates of dispersal. Moreover, the lack of clear establishment or spread from our third, significantly smaller, release area (only 0.11 km^2^) is consistent with the prediction for bistable dynamics that releases must be conducted over sufficiently large areas to initiate spatial spread.

Our likelihood analyses also quantify significant heterogeneity in rates of spatial spread that is apparent from our graphical representations. We attempt to link this heterogeneity to easily measured habitat variables. Heterogeneity in host population density is expected to strongly influence *Wolbachia* invasions subject to bistable dynamics, especially affecting wave speed and potentially restricting the extent of spread [20]. Even if *Ae*. *aegypti* disperse equally in all directions, heterogeneities in population density produce asymmetries in net migration. This asymmetry accelerates spread from high-density patches to low-density patches and decelerates—or halts—spread out of low-density patches [20]. Habitat variables such as shade, yard condition, and abundance of oviposition sites have been correlated with *Ae*. *aegypti* abundance [41,51]; the frequency of *Wolbachia* infection within the release zone in Gordonvale, Queensland, was higher in neighbourhoods with more brick and screened houses, which are associated with lower *Ae*. *aegypti* abundance [32]. This motivates our attempts to understand patterns of local spread by inferring local densities from easily measured habitat variables. However, the variables we assessed did not predict observed heterogeneities in spread beyond the release zones.

## Results

### Mosquito collections and abundance

**Fig 1:**
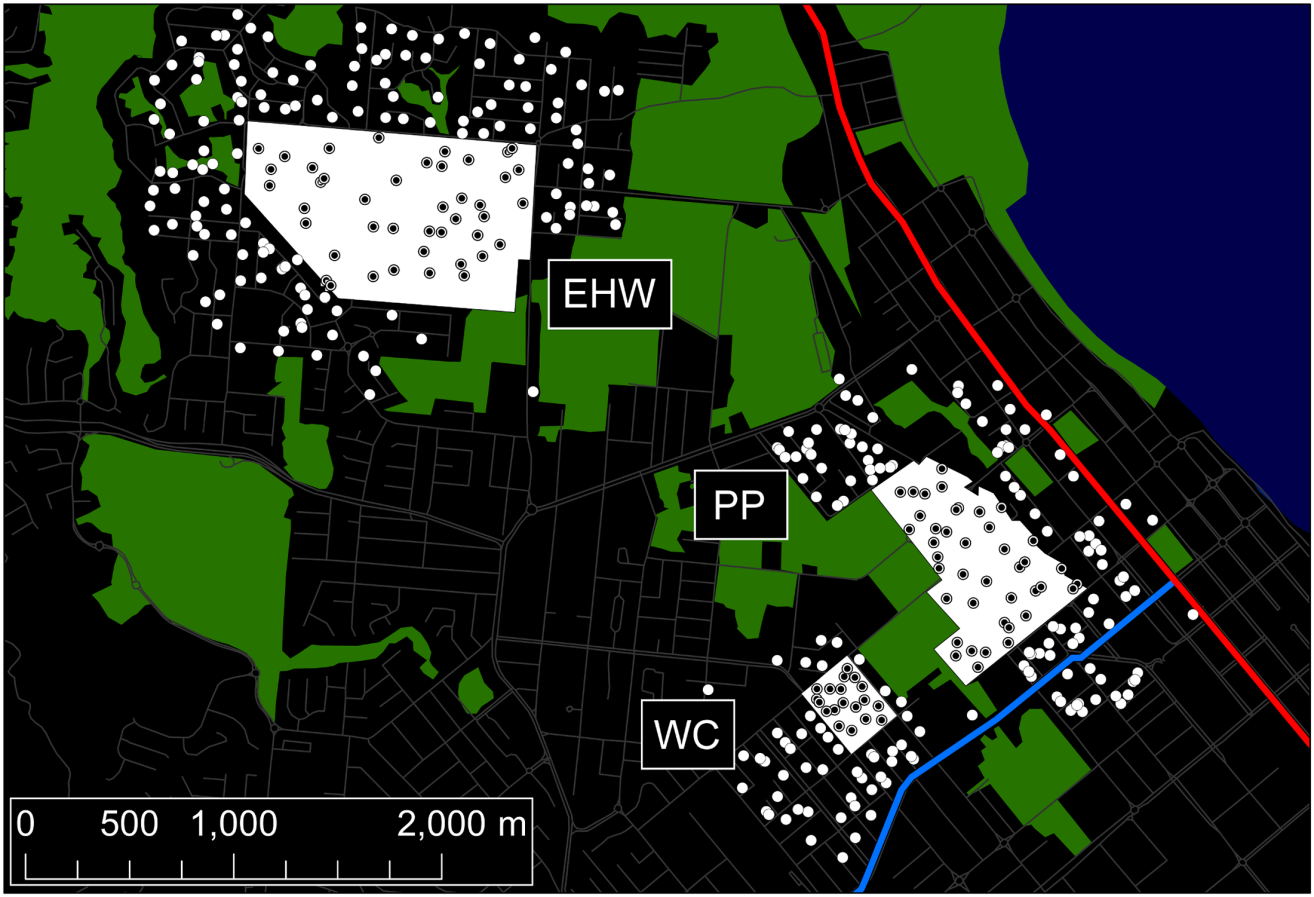
Release zone locations in Cairns. The 3 release areas are Edge Hill/Whitfield (EHW), Parramatta Park (PP), and Westcourt (WC). Locations of the 2 major highways, Mulgrave Road and Captain Cook Highway, are indicated in light blue and red, respectively. Locations of onsite traps (traps within the release zones) are plotted as black dots within white circles, and offsite traps (outside the release zones) are plotted as white circles. (The underlying road network is derived from "Australia Oceania Continent Roads," made available by MapCruzin.com and OpenStreetMap.org under the Open Database License [https://opendatacommons.org/licenses/odbl/1.0/].)

Fig 1 shows the 3 areas in Cairns, Queensland, where Eliminate Dengue staff released *Ae*. *aegypti* adults infected with the *w*Mel strain of *Wolbachia* between January 10th and April 24th, 2013. The release zones, located in the suburbs of Edge Hill/Whitfield (EHW), Parramatta Park (PP), and Westcourt (WC), were within 2 km of each other and encompassed 0.97 km^2^, 0.52 km^2^, and 0.11 km^2^, respectively. Mosquitoes were released evenly throughout each release zone at weekly intervals. Total BG-Sentinel trap collections for EHW, PP, and WC are summarised in S1 Table. Our collections continued for about 2 years and are summarized in 4 time intervals. The first dry season D1 (May 2013–October 2013), began immediately after the releases, followed by the first wet season W1 (November 2013–April 2014), the second dry season D2 (May 2014–October 2014), and the second wet season W2 (November 2014–April 2015).

**Fig 2:**
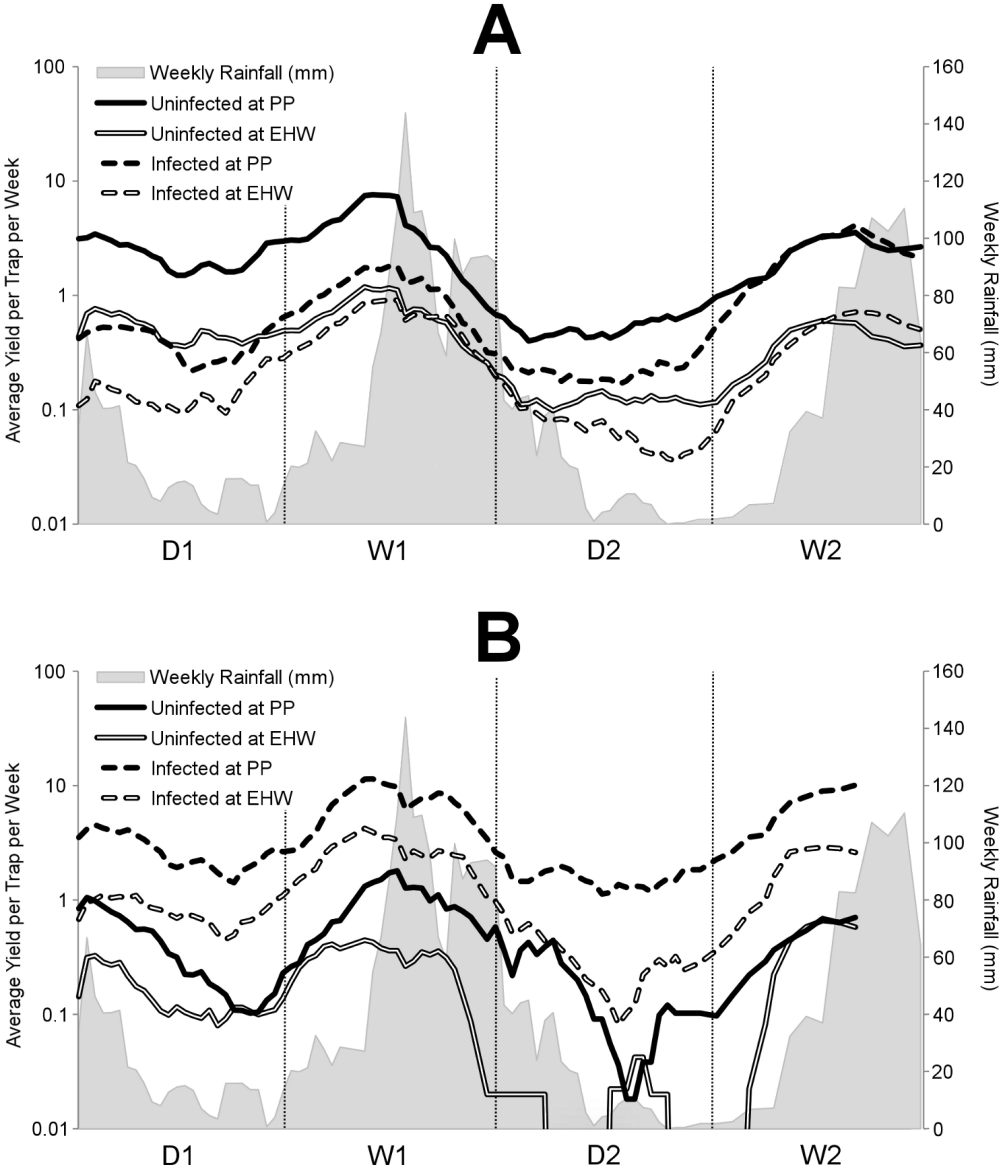
Average trap captures for infected and uninfected *Ae*. *aegypti*. *Ae*. *aegypti* caught offsite (A) and onsite (B) are graphed atop weekly Cairns rainfall. Yields and rainfall are smoothed using a moving average of the 5 most recent observations. Trap yields are plotted on a logarithmic scale to show comparative rates of change. After accounting for the seasonal trend in abundances, uninfected mosquito yields offsite (A) decreased over time at both Parramatta Park (PP) and Edge Hill/Whitfield (EHW). Offsite yields of infected mosquitoes increased at PP towards the end of the study but remained relatively constant at EHW. Onsite (B) yields of infected mosquitoes remained relatively constant at EHW and PP, while uninfected mosquito yields decreased heavily in the second dry season (D2) but recovered in the following season.

Weekly trap yields at EHW and PP decreased progressively from the onset of each dry season but rose again sharply at the beginning of each wet season (Fig 2). Mosquitoes were caught in consistently higher numbers at PP than at EHW (two-tailed Student *t* test: *P* < 0.001), and onsite traps (traps within the release zone) collected mosquitoes at a faster rate than offsite traps (traps outside the release zone; two-tailed Student *t* test: *P* < 0.001). When accounting for seasonal changes, yields of uninfected mosquitoes caught in offsite traps at both sites tended to decrease over time (Fig 2 panel A). At PP, there was a corresponding increase in infected mosquito numbers, while at EHW, infected mosquito yields were relatively consistent throughout.

Among onsite traps, yields of infected mosquitoes were consistent with seasonal expectations and were stable over time (Fig 2 panel B). The higher local infection frequencies onsite might lead to an assumption that uninfected mosquito numbers would decline more rapidly than those offsite, but this was not observed, with uninfected mosquito yields at both sites increasing sharply in W2. This proliferation was particularly surprising considering the 2-month-long periods at EHW in the previous season, D2, during which no uninfected mosquitoes were caught onsite (Fig 2 panel B).

### *Wolbachia* frequencies: Onsite

EHW and PP were both invaded quickly, and by the time releases had finished, *Wolbachia* infection frequencies within each release zone had reached *p* = 0.85 (S1 Fig). Following the final releases, *p* remained relatively stable and near fixation within each release zone. However, in W2, onsite *p* at EHW dropped from 0.96 to 0.84, the lowest recorded since monitoring began. Considering Fig 2 panel B, it appears that this was due to neither imperfect maternal transmission of *Wolbachia* [26] nor increased mortality among infected mosquitoes, as their numbers increased to levels similar to those observed in W1. Rather, a sudden influx of uninfected mosquitoes seems most plausible. Averaged across all 4 seasons, 0.88 of mosquitoes within the EHW release zone were infected, while 0.90 were infected at PP.

The WC release zone was invaded as quickly as EHW and PP. However, beginning in September 2013, onsite *p* dropped sharply to *p* < 0.7, after which frequencies fluctuated. While onsite *p* never dropped below any plausible value for $\hat{p}$, at no point did the invasion at WC exhibit either the near-fixation values of *p* or the temporal stability observed at both EHW and PP (see figures below and compare panel C of S2 Fig with panels A and B).

### Spread of *w*Mel at EHW and PP but apparent collapse at WC: Graphical analysis

**Fig 3:**
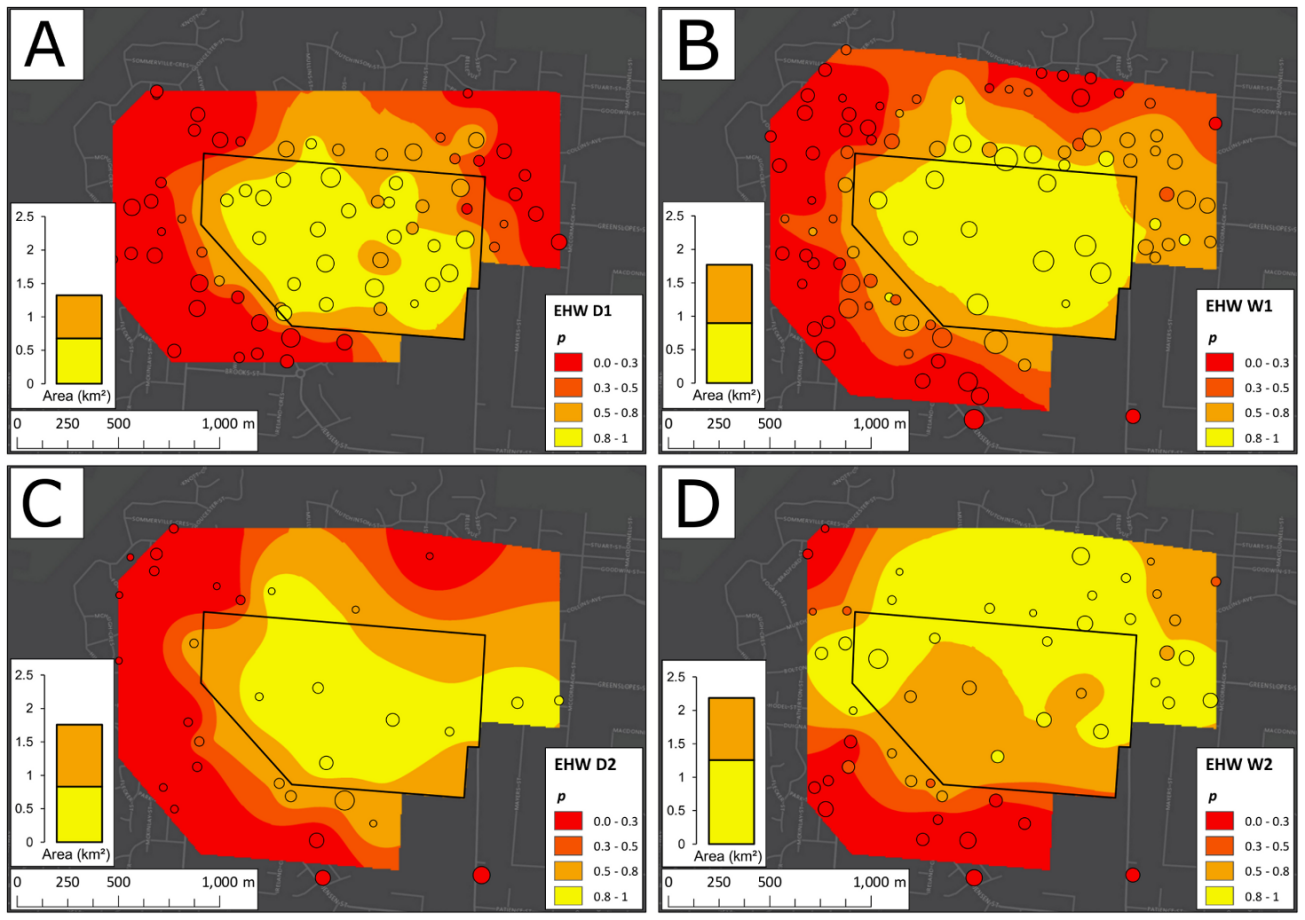
Ordinary Kriging of infection frequency (*p*) among traps at Edge Hill/Whitfield (EHW). Kriging (spatial averaging) was performed using an exponential semivariogram model and a 24-point, nearest-neighbour search function for the first dry season (D1) (panel A), the first wet season (W1) (B), the second dry season (D2) (C), and the second wet season (W2) (D). The central black polygon depicts the release zone. Trap locations are plotted as circles atop each Kriging map and are sized by a logarithmic function of the trap yield from each season. To the left of each Kriging plot, a stacked column chart displays the areas enclosed by the *p* > 0.8 and *p* > 0.5 contours. Although a contraction took place in D2, the area increased in W2, despite infection frequency decreasing in several sites within the release zone.

**Fig 4:**
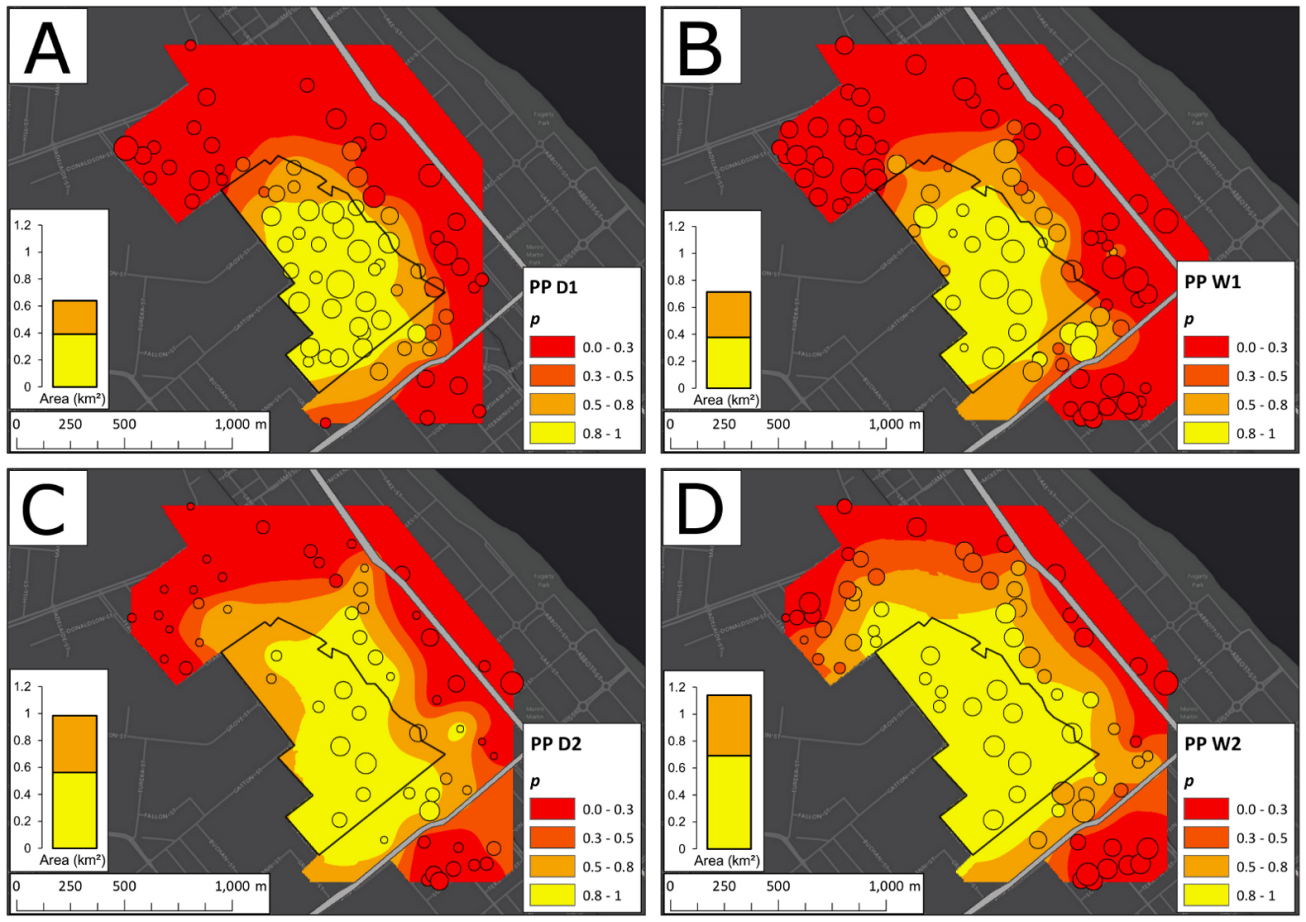
Ordinary Kriging of infection frequency (*p*) among traps at Parramatta Park (PP). Kriging was performed as in Fig 3 for the first dry season (D1) (A), the first wet season (W1) (B), the second dry season (D2) (C), and the second wet season (W2). The central black polygon depicts the release zone. Trap locations are plotted as circles atop each Kriging map and are sized by a logarithmic function of the trap yield from each season. To the left of each Kriging plot, a stacked column chart displays the areas enclosed by the *p* > 0.8 and *p* > 0.5 contours, showing a constant increase in the invaded area over time.

**Fig 5:**
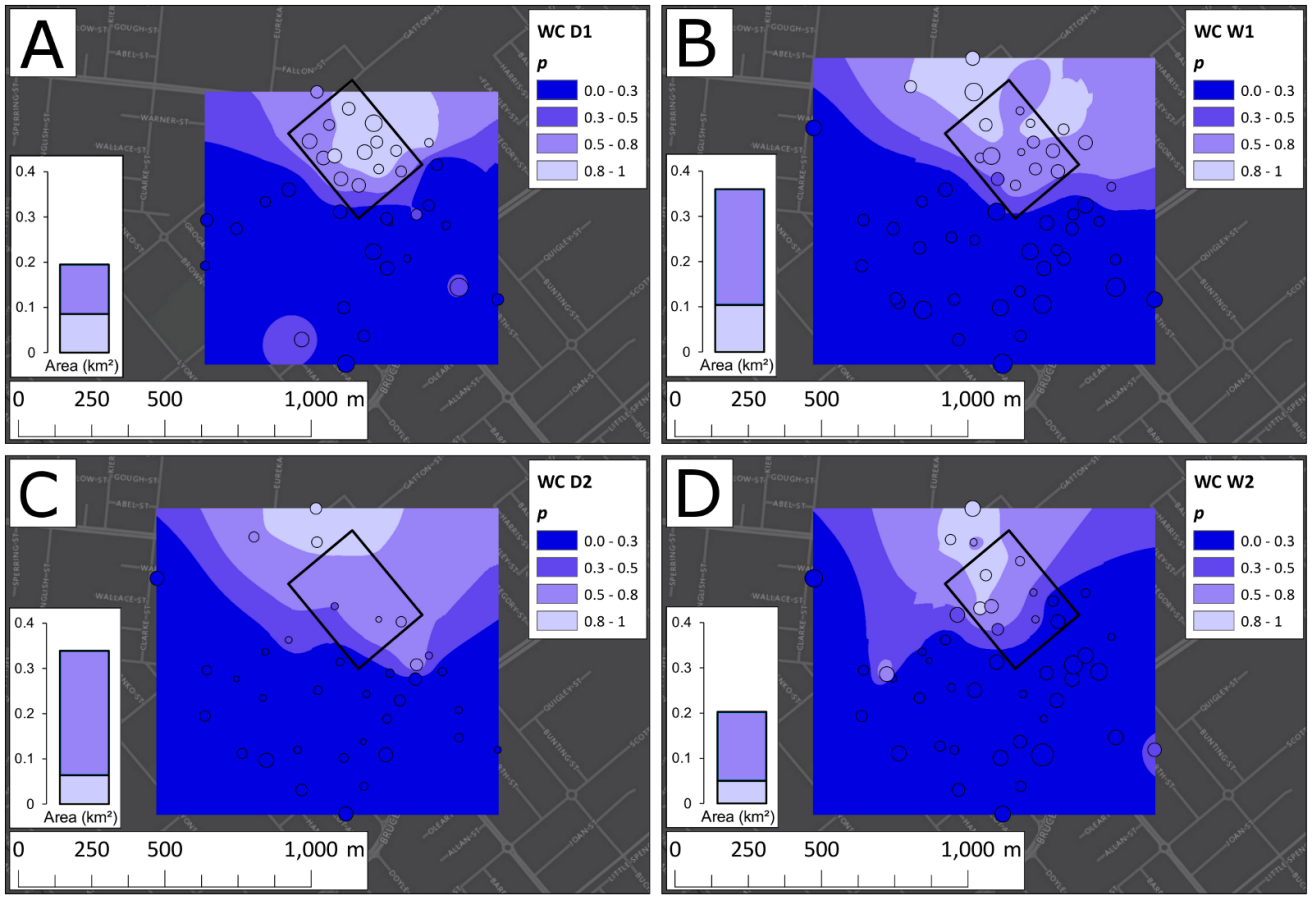
Ordinary Kriging of infection frequency (*p*) among traps at Westcourt (WC). Kriging was performed using an exponential semivariogram model and a 16-point nearest-neighbour search function for the first dry season (D1) (A), the first wet season (W1) (B), the second dry season (D2) (C), and the second wet season (W2). The central black polygon depicts the release zone. Trap locations are plotted as circles atop each Kriging map and are sized by a logarithmic function of the trap yield from each season. To the left of each Kriging plot, a stacked column chart displays the areas enclosed by the *p* > 0.8 and *p* > 0.5 contours, showing a decline in the area of infection following W1.

The changes of *p* with time at EHW, PP, and WC between 7 May 2013 and 30 April 2015 are displayed in Figs 3, 4 and 5, respectively, along with trap locations and yields. The plots, based on spatial averaging (ordinary Kriging as described in the Methods section, performed using ArcMap 10.2.2 [52]), show considerable seasonal heterogeneity in the spatial structure of the invasions at EHW, PP, and WC.

At EHW (Fig 3) after D1, the infection was confined largely to the north and northeast, but by the end of W1, the invasion had spread to the east, northeast, and southwest. This pattern persisted through D2, with a small retraction in the north and expansion in the east, though for this season, Kriging was affected by a small sample size (*N* = 31). Kriging on W2 trap data demonstrated 3 main shifts from this pattern: the continued expansion to the north, northeast, and east; the successful invasion of the west; and the apparent reduction in *p* from *p* ≥ 0.8 to 0.65 ≤ *p* ≤ 0.8 at 3 traps in the centre of the release zone.

At PP (Fig 4), spread through D1 was confined mostly to the southeast, from the edge of the release zone up to Mulgrave Road. In the following season (W1), infected mosquitoes were found south across Mulgrave Road and north of the release zone. The infection persisted south of Mulgrave Road but only at below-threshold (*p* ≤ 0.3) frequencies. Over D2, the invasion expanded in range, with high frequencies observed in the north and the southeast and moderate frequencies in the northwest.

At both EHW and PP, the area covered by the infection tended to increase over time (Figs 3 and 4; summarized in panels A and B of S2 Fig), except for PP in W1, in which the area within the *p* ≥ 0.8 contour decreased by 3% from D1, and for EHW in D2, in which the area within the *p* ≥ 0.8 and *p* ≥ 0.5 contours decreased by 7% and 1%, respectively. Nevertheless, from D1 to W2, the area enclosed by the *p* ≥ 0.8 contours grew by 85% at EHW and 77% at PP.

At WC (Fig 5; S2 Fig panel C), establishment or spread of the infection was not observed. Following D1, the *Wolbachia* invasion failed to expand to the south or west of the release zone. Within the release zone, a gradual retreat of the *p* ≥ 0.8 contour was observed, with several onsite traps in W2 registering *p* ≤ 0.3. The area covered by the infection at WC reached a peak at W1, but by W2 the area enclosed by the *p* ≥ 0.8 and *p* ≥ 0.5 contours had decreased by 52% and 44%, respectively, from this maximum (S2 Fig panel C). *Wolbachia* also failed to spread from WC into PP (or vice versa), but these areas are separated by parkland, which is likely to act as a barrier to movement and prevents ongoing monitoring there.

### Spread of *w*Mel at EHW and PP: Heuristic analysis of graphical data summaries

The time interval from D1 to W2 is around 1.5 years, which can be approximated as 15 generations, assuming about 10 generations per year (explained below), or simply viewed as 548 days. When containers are initially colonised and food is available for larvae, developmental time is likely to be rapid at 7–10 days. However, larval populations can rapidly exceed the carrying capacity of the container and its food source (typically leaves), and development is then slowed to 20–50 days [53]; these variable conditions produce a range of adult body sizes that is typically found in field samples from Cairns [54]. If we assume an intermediate value of 20 days in the field, along with time for adult maturation to mating and blood feeding (2–3 days post eclosion), blood-meal digestion and egg formation and oviposition (4 days), and egg embryonation (3 days) [55], this adds another 10 days of adult and egg developmental time. In Cairns, a cooler winter period will lengthen developmental periods, while dry periods delay hatching. Overall, 10 generations per year is likely to be a reasonable estimate.

S2 Fig provides approximations for the areas covered by *w*Mel in different seasons after the releases. For EHW, the area covered in which *w*Mel has at least frequency 0.5 is about 1.3 km^2^ in D1, and this rises to about 2.2 km^2^ in W2. We can calculate wave speed per generation (assuming 10 generations a year) or per day using alternative geometric approximations described in the Methods section: approximation 4 assumes a circular release area, approximation Eq (5) assumes a rectangle (for which we approximate parameter *y* = 2 [i.e., a release area twice as long as wide]), or approximation 6 assumes a rectangle in which spread does not occur (or is not monitored) in one direction. (Note that very little spread occurred to the south at EHW.) The resulting estimates of wave speed per day are, respectively, *c*_d_ = 0.35 m per day, 0.31 m per day, and 0.45 m per day. If we assume 10 generations per year (and so 15 generations separating D1 from W2), the corresponding wave speeds per generation are: *c* = 12.9, 11.2, and 16.6 m/gen.

Assuming dispersal parameter *σ* ≈ 100 m/(gen)^1/2^ and unstable equilibrium $\hat{p}\approx 0.3$ (see Turelli and Barton [31]), the cubic diffusion approximation for wave speed (see Eq 2), $c=\sigma (½–\hat{p})$, predicts roughly 20 m/gen. As discussed in the context of our likelihood analyses below, the discrepancy between the estimated speeds and this analytical prediction can be resolved by assuming longer generations, a higher unstable point, and/or long-tailed dispersal [31].

For PP, the area covered in which *w*Mel has at least frequency 0.5 is about 0.65 km^2^ in D1, and this rises to about 1.17 km^2^ in W2. Using our geometric Models 4, 5 and 6 (with *y* = 2, as for EHW), the resulting estimates of wave speed per day are, respectively, *c*_d_ = 0.28 m per day, 0.25 m per day, and 0.37 m per day. If we assume 10 generations per year (and so 15 generations separating D1 from W2), the corresponding wave speeds per generation are: *c* = 10.4, 9.0, and 13.4 m/gen. The speed estimates for PP are systematically smaller than for EHW. As discussed in the Methods section, both wave speed and wave width (describing the distance over which infection frequencies change appreciably) are proportional to average dispersal distances. Thus, slower wave speed is expected if the higher adult densities observed at PP versus EHW translate into a more desirable habitat and consequently smaller average dispersal distances (lower *σ*). Consistent with this, we find a sharper wave at PP as quantified by smaller average distances between the 0.3 and 0.8 contours at PP than EHW; these distances average 326 m at EHW and only 252 m at PP.

### Spread of the infection at EHW and PP: Likelihood analysis

Our likelihood analyses are independent of the graphical summaries produced by Kriging. They rely on an approximate description of the expected shape of local spread and/or collapse (see Eq 7 in the Methods section). We present several successive analyses that summarize the rate and pattern of spatial spread of *Wolbachia* at EHW and PP. Our summaries focus on 2 statistics: wave width and wave speed. We start by analysing the data averaged over space and time, then present more detailed analyses that document heterogeneous spread. We begin by analysing the data assuming that observed frequencies deviate from deterministic expectations only because of binomial sampling variation. We then use a more complex probability model that accounts for additional sources of heterogeneity. Finally, we explicitly test for directional heterogeneity in rates of spread, as documented visually in Figs 3 and 4. The details of the likelihood analyses are relegated to S1 Text.

#### Analysis of pooled data

S3 Fig panel A shows the EHW data through time, averaged over time and space. As described in the Methods section, time is measured in days from 3 October 2012. Releases began on 10 January 2013 (day 99) and ended on 18 April 2013 (day 197). We averaged the data over 9 time intervals with boundaries at 90, 110, 120, 150, 250, 300, 400, 550, 700, and 900 days (with midpoints of 100, 115, 135, 200, 275, 350, 475, 625, and 800 days). For each time interval, we averaged the frequency data over 100-m intervals, with distance (for each trap) relative to the edge of the release area (r* = 0). S3 Fig panel B shows the Gaussian/logistic Model 7 with the maximum likelihood estimates of the parameters *r*_0_ describing the position of the wave and *w* describing the wave width, assuming only binomial sampling variation in the infection frequencies. S3 Fig panels C and D show the comparable results for PP. The first 2 curves in S3 Fig panels B and D, centred on days 100 and 115, document the initial rise of the infection within the release area, just after the releases began on day 99. Field releases ended on day 197, and the green lines in S3 Fig panels B and D (centred on day 200) show the infection clearly spreading beyond the release areas.

**Fig 6:**
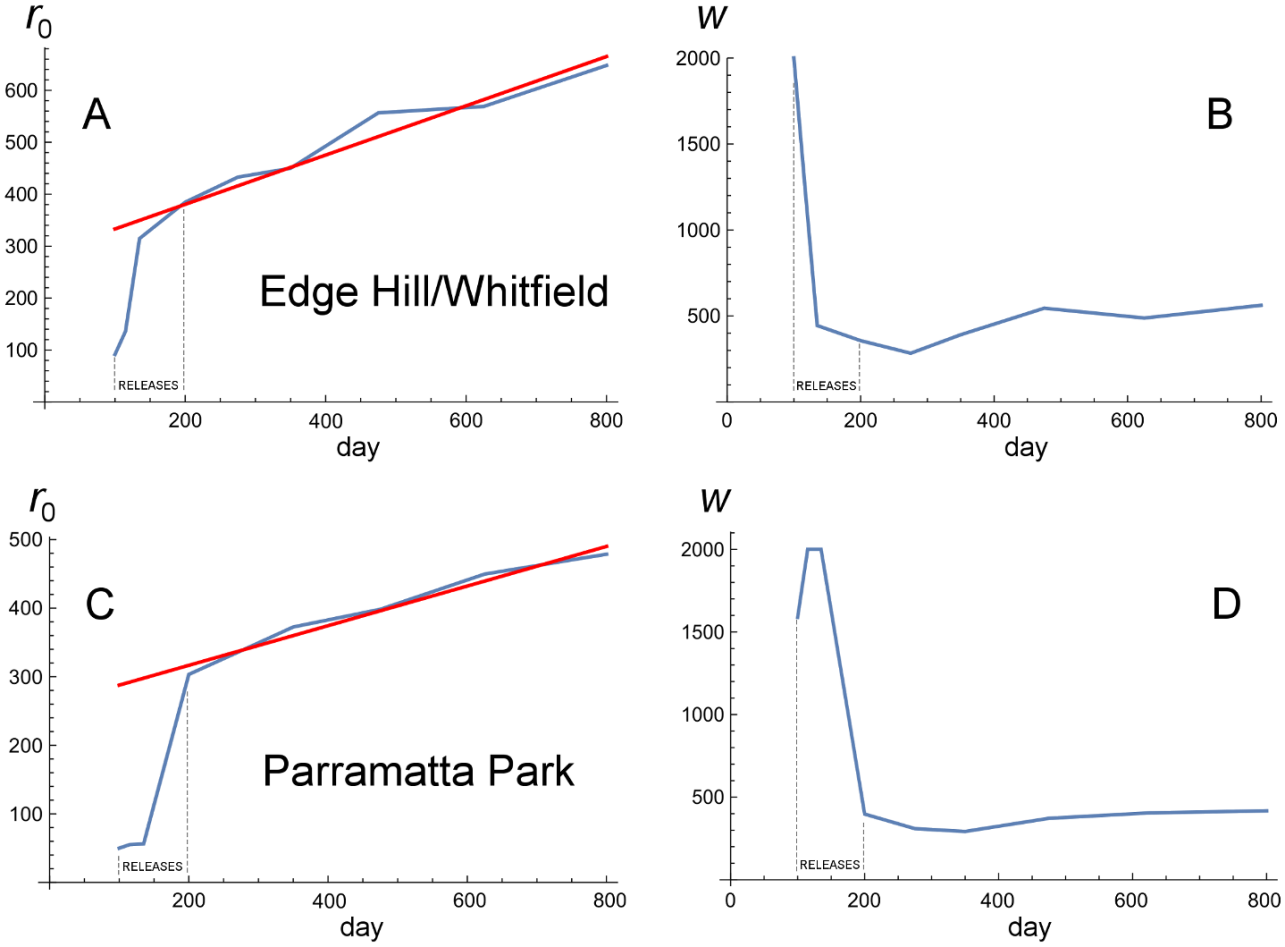
Estimating rates of spatial spread and wave width for Edge Hill/Whitfield (EHW) and Parramatta Park (PP). Panels A and B plot the estimates of *r*_0_ and *w* (from S2 Table) through time for EHW; panels C and D show the estimates for PP. The *x* axis in each panel represents days; the releases began on day 99 and ended on day 197. The slopes of the fitted regression lines imply wave speeds of *c*_d_ = 0.474 m per day at EHW and *c*_d_ = 0.289 m per day at PP. See the text for discussion and interpretation.

S2 Table provides the maximum likelihood estimates of *r*_0_ and *w* of the Gaussian/logistic Model 7 for each time interval with the corresponding likelihood Log(*L*). Note that *r*_0_ ≥ 0 is measured relative to the centre of the release areas, so that a value near 340 m (220 m) corresponds to the edge of the EHW (PP) releases. When *r*_0_ << *w*, the Gaussian/logistic model approximates a Gaussian, centred at 0, as illustrated by the figure in our Methods section. Fig 6 plots the estimates of *r*_0_ and *w* against time (in days) over the 9 time intervals.

For EHW, the increase in *r*_0_ (wave position) becomes roughly linear with time after the first 2 intervals (i.e., after day 120), and the estimated values of *w* (wave width) settle down to approximate constancy. In this initial phase of spread, *r*_0_ ~ *w* and the fitted model approximates a logistic. Dropping the first 2 time intervals, the regression of *r*_0_ on time has slope *c*_d_ = 0.474 m per day. This estimate is broadly consistent with our heuristic graphical wave-speed estimates of 0.31–0.45 m per day. The mean wave width (again, dropping the first 2 intervals) is 439 m.

For PP, the increase in *r*_0_ becomes roughly linear with time after the first 3 intervals (i.e., after day 150), with *r*_0_ ~ *w*, and the fitted model approximates a logistic. Dropping the first 3 time intervals, the regression of *r*_0_ on time has slope *c*_d_ = 0.289 m per day at PP; the mean wave width is 366 m. Again, the likelihood estimate of wave speed is comparable to our heuristic graphical estimates of 0.25–0.37 m per day. Moreover, our statistical results agree qualitatively with our graphical analyses in showing slower spatial spread at PP than EHW, with the slower speed accompanied by a sharper cline in frequencies (i.e., smaller *w*).

Before comparing these results to our theoretical predictions, we consider 2 statistical refinements, whose methods are described in S1 Text. The likelihood analysis summarized in Fig 6 assumes that binomial sampling is the only source of variation in infection frequencies observed at fixed distances from the release areas. It also fits frequency data averaged over time and space. In S1 Text section 1.1, we first generalize our statistical model to allow for nonbinomial variation, implicitly accounting for factors such as environmental heterogeneity that contribute to differences in infection frequencies among sampling locations equidistant from the release areas. The additional stochasticity is summarized by a parameter *F*, where *F* > 0 accounts for greater-than-binomial variation. The likelihood analyses for EHW and PP incorporating this factor appear in S3 and S4 Tables, respectively. Our second refinement fits the data directly without spatial or temporal averaging. This involves estimating a constant, denoted *R*, which describes how far the wave has moved from the centre of each release area when approximately linear spread was observed (see S1 Text section 1.2 for details). Table 1 shows that our likelihood estimates of wave speed, *c*_d_ (measured as meters per day), and wave width, *w*, are relatively insensitive to these more refined analyses.

**Table 1:** Maximum likelihood estimates (MLEs) of parameters describing spatial spread.

Method or model	Edge Hill/Whitfield			Parramatta Park		
	*R*	*c*_d_	*w*	*R*	*c*_d_	*w*
MLE (no pooling, *F* fixed)	282	0.51	469	252	0.3	384
Support limits	(263, 302)	(0.46, 0.55)	(444, 495)	(235, 270)	(0.27, 0.34)	(369, 401)
MLE (pooling, *F* variable)	__	0.47	456	__	0.29	384
MLE (pooling, *F* fixed)	__	0.48	454	__	0.29	390
MLE (pooling, *F* = 0)	__	0.47	439	__	0.29	366

Summary of MLE of parameters describing spatial spread with and without data pooling; *R* is a constant describing the distance from the centre of the release area at which approximately linear spread was observed, *c*_d_ estimates wave speed as m per day, and *w* estimates wave width in meters.

#### Analyses of heterogeneous spatial spread at EHW

Fig 3 shows the heterogeneous spread of *w*Mel at EHW, with more rapid spread to the north and east than to the south and west. Here we summarize a likelihood analysis that quantifies the heterogeneity. The details of the analysis are provided in S1 Text section 1.3. For simplicity, we divided the samples into 4 equal-angle triangular sectors, centred on the middle of the release area. We fit Model 7 to the pooled data from each sector separately and sought the orientation of the sectors that produced the best fit to the data (allowing for additional nonbinomial variance as discussed above). This allows us to estimate 4 separate wave speeds and apply a likelihood test for heterogeneity. The results of the analysis, demonstrating statistically significant spatial heterogeneity in the rates of spread, are summarized in S5 Table and illustrated in S4 Fig.

### Collapse of the introduction at WC: Likelihood analysis

**Fig 7:**
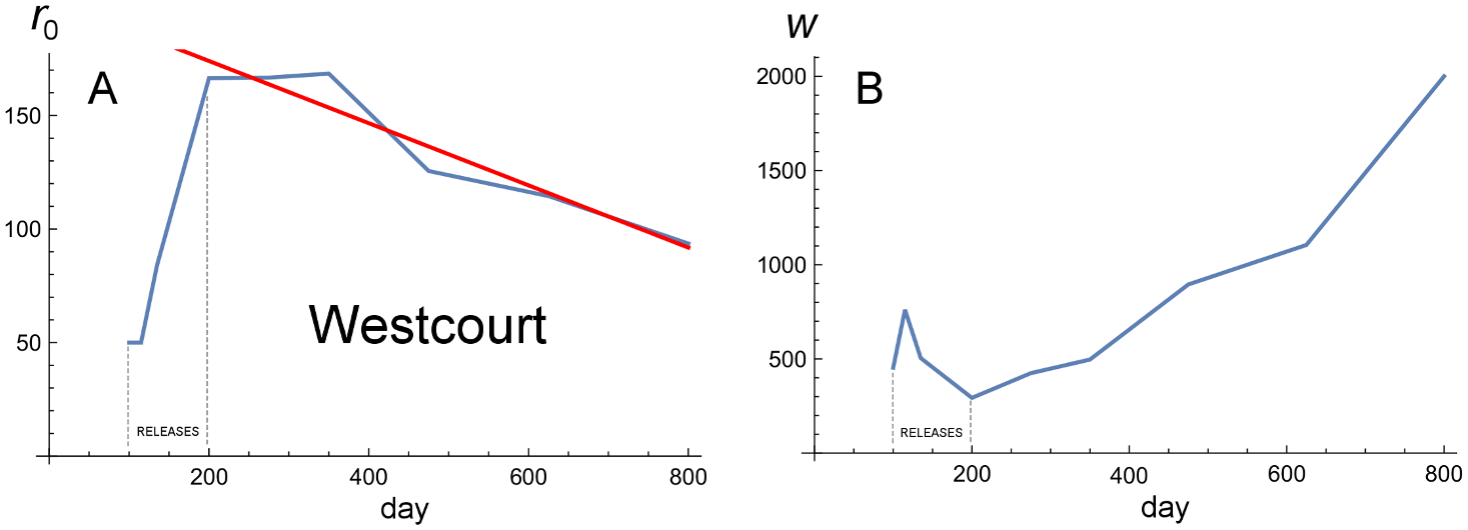
Likelihood analysis of the Westcourt (WC) data. Panels A and B plot the estimates of *r*_0_ and *w* (from S6 Table) through time for WC. See the text for discussion and interpretation.

Fig 5 illustrates the slow collapse of the *w*Mel introduction at WC. As shown in S2 and S5 Figs, in contrast to the rising infection frequencies outside the release zones at EHW and PP, *p* initially rises then slowly falls near the WC release. A likelihood analysis of the pooled data, analogous to those presented in Table 1, S3 Fig and Fig 6, supports this conclusion. The details of the analysis are given in S6 Table, with the results graphically summarized in Fig 7. Unlike the steady outward movement of the wave shown at EHW and PP, with the wave widths stabilizing at values near 400 m, Fig 7 shows that the estimated location, *r*_0_, of the “wave” at WC retreats through time, while the wave width, *w*, steadily increases, corresponding to slow collapse of the *w*Mel introduction.

### Comparison of observed spread at EHW and PP to theoretical predictions

From our likelihood analyses, the wave speed *c*_d_ is approximately 0.5 m per day (186 m per year) at EHW with wave width *w* about 460 m. In contrast, we find a slower moving and sharper wave at PP with *c*_d_ approximately 0.3 m per day (110 m per year) and wave width *w* about 380 m. These estimates are broadly consistent with our heuristic approximations (from Eqs 4–6) obtained from the Kriging plots in Figs 3, 4 and 5. As demonstrated by Turelli and Barton [31], even with fast local dynamics and long-tailed dispersal, we can accurately approximate average local dispersal as *σ* = *w*/4 m/(gen)^1/2^ (Eq 2). From this we infer *σ* ≈ 115 m/(gen)^1/2^ at EHW; in contrast, we obtain *σ* ≈ 95 m/(gen)^1/2^ at PP. Given that the support intervals for the estimates of *w* at EHW and PP do not overlap (Table 1), we expect these results reflect differences in local dispersal. Given that PP has consistently higher population densities, this difference may reflect less dispersal in a habitat where mosquito densities are higher. However, this needs further testing against alternative hypotheses, such as more dispersal barriers surrounding the PP versus the EHW release areas. It is notable that both the EHW and PP estimates of dispersal are consistent with values obtained from release–recapture experiments (reviewed in [31]).

If we assume that the wave speed follows the cubic diffusion approximation $c=\sigma (½–\hat{p})$, per generation and that generations are *T* days long, we can in principle reconcile observed wave speeds with expected wave speeds at each release site by choosing $\hat{p}$ and *T* appropriately, namely

$$T=\sigma \left(\frac{1}{2}-\hat{p}\right)/c_{d},$$ (1)

where *σ* is the local dispersal estimate and *c*_d_ is the observed wave speed per day. For instance, if we assume that at both EHW and PP, $\hat{p}=0.3$, the observed and expected wave speeds can be reconciled if we assume that *T* = 46 days for EHW, whereas *T* = 63.3 days for PP. Given that population densities are higher for PP, increased crowding may indeed produce longer generation times [53]. These times are systematically larger than our conjecture of 10 generations per year, which we supported by an informal data review above.

These inferences assume that the cubic-diffusion prediction for wave speed ($c=\sigma (½–\hat{p})$ per generation) is accurate for these field populations. However, as shown by Turelli and Barton [31], long-tailed dispersal with fast local frequency dynamics (as expected with complete cytoplasmic incompatibility, corresponding to *s*_h_ = 1 in the models of [31]), can slow the expected wave speed by 20%–40% below the cubic-diffusion prediction. If the expected wave speed is reduced by 30%, the observed wave speeds match the modified expectations with generation times reduced to 32.2 and 44.3 days at EHW and PP, respectively. These times are closer to our conjecture of 10 generations per year. In general, there seems to be reasonable quantitative agreement between the slow observed wave speeds and the predictions of simple models using parameter values that are consistent with the poorly known field biology of *Ae*. *aegypti* and the deleterious fitness effects of wMel in *Ae*. *aegypti*. Despite many caveats, including uncertainty about parameter values and the imprecise meaning of the one-dimensional unstable point $\hat{p}$ for populations with overlapping generations and complex ecology [27], the observed spread rates at EHW and PP are clearly consistent with approximation Eq (1) using plausible estimates of dispersal distance, the unstable point, and generation time.

### Comparison of apparent collapse at WC to theoretical predictions

In contrast to EHW and PP, the releases at WC did not lead to clear establishment and certainly did not produce spatial spread (see Figs 5 and 7). Turelli and Barton [31] provide conditions on minimum release areas (and maximum dispersal distances) consistent with spatial spread, allowing for long-tailed dispersal and rapid local dynamics. We expect that $\hat{p}\approx 0.25–0.3$ and *σ* ≈ 100 m/gen^1/2^. If these parameter estimates are accurate, the release area at WC is likely to be just below the minimum needed to produce successful local establishment and spread (see Table 2 of [31]). Moreover, the fact that the apparent collapse at WC is extremely slow is consistent with the slow dynamics expected near that critical size threshold for wave-establishing releases [31]. Overall, the bistable dynamics of *w*Mel in *Ae*. *aegypti* will impose some minimum release size, and only WC is near a plausible minimum. To rigorously test the minimum-release-area predictions of Barton and Turelli [20] and Turelli and Barton [31], several more replicate releases in small areas would be needed.

## Discussion

Our data demonstrate that *w*Mel can be stably established locally within urban areas surrounded by uninvaded but suitable habitat. Hence, stable population replacement is not limited to small isolated habitats such as those where the initial releases and establishment of *w*Mel in *Ae*. *aegypti* took place (cf. [14]). Moreover, the temporal increase in infection frequency within the EHW and PP release zones was comparable to that seen in the isolated areas. In contrast, the smallest release area, WC, did not show stable invasion. This suggests that there is little impediment to the local establishment of *Wolbachia* in urban areas, provided the releases are conducted over sufficiently large areas (e.g., on the order of 0.5 km^2^ when dispersal distances are comparable to those in Cairns [31]). These findings highlight the feasibility of patchy releases across large cities, suggesting that area-wide replacement can be produced gradually, with patchy releases complemented by natural local spread. At EHW and PP, the area in which *Wolbachia* persists at high frequency roughly doubled after 2 years (Figs 3 and 4).

The failure of *w*Mel to establish and spread at WC seems attributable to the small area of the release zone, as the habitat conditions in and around WC are similar to EHW and PP. This is consistent with mathematical predictions concerning the minimum release zone radius, *R*_crit_ [20,31]. Based on the wider advancing wave front seen at EHW versus PP, we infer greater average dispersal distance at EHW (which is likely to provide fewer feeding and breeding opportunities than PP). Mosquito dispersal differences probably explain the faster spread observed at EHW versus PP. In contrast, the slow temporal and spatial dynamics of local infection frequency at WC suggests that 0.11 km^2^, the area of the WC release zone, may be very close to the minimum size needed to initiate spread, at least for the levels of dispersal typical of Cairns. When contrasted against the successful spread at PP, we conclude that the critical release area under Cairns conditions is somewhere between 0.11 km^2^ and 0.52 km^2^. In tropical regions that support denser *Ae*. *aegypti* populations, we expect lower dispersal distances. This would allow successful local establishment using smaller release areas, but spatial spread would also be expected to be even slower than the 100–200 m per year observed at EHW and PP.

The heterogeneity in both the speed and patterns of the spatial dynamics at EHW and PP suggests that local environmental factors greatly influence the spread of *Wolbachia* transinfections (such as *w*Mel in *Ae*. *aegypti*) that produce significant fitness costs. Spread at each site exhibited strong spatial structure throughout the study, and the structure persisted across the monitoring period. Areas that were easily invaded during the first dry season after the releases (D1, see Figs 3 and 4) generally stayed invaded in successive seasons, and the autocorrelation among mosquito numbers and infection frequencies increased as the study progressed (S7 Table). The invasion spread well beyond the initial release zones at EHW and PP, and our likelihood analyses (Fig 6) suggest that slow but steady spread would continue in the absence of further releases until significant barriers to dispersal are encountered.

Barriers to spread can include both barriers to *Ae*. *aegypti* dispersal and variation in *Ae*. *aegypti* population density [20]. At PP, the invasion spread south from the release zone immediately but never established to a high frequency south of Mulgrave Road. Nevertheless, infected mosquitoes were caught at low frequencies south of Mulgrave Road from season W1 onwards. These observations are consistent with the demonstration in Trinidad that roads represent partial barriers to *Ae*. *aegypti* dispersal [43]. At the very least, such barriers slow wave propagation [20]. It remains unclear whether Mulgrave Road provides a sufficient barrier to stop the wave of *Wolbachia*, as is the case of the Bruce Highway at Gordonvale. There, *Wolbachia* have failed to invade an area adjacent to the 2011 release zone for several years, despite persistent migration across the highway [31]. Other evidence from mark-release experiments and genetic studies have pointed to potential barriers (roads, rivers, forests) to movement of *Ae*. *aegypti* at a local scale [43].

In W2 at EHW, there was an apparent drop in *p* in the southern half of the release zone. This was unexpected given that in previous seasons traps in this region had recorded *Wolbachia* frequencies close to fixation. It appears that the drop was due to a sudden increase in uninfected mosquito numbers onsite, which may represent the hatching of dormant uninfected eggs or an early influx of uninfected mosquitoes from an external source at the start of W2. One possibility is that *Wolbachia* infected larvae experienced a fitness cost under high-stress conditions prevailing at that time; such costs have been recently documented under stressful conditions that produce a range of adult sizes [19,56] similar to those seen under field conditions [54], even though earlier studies suggested only modest fitness costs associated with *w*Mel [11,16]. The openness of the EHW study area may also make it more vulnerable to reinvasion, as immigration into the release zone was possible from 360° of the surrounding area. In contrast, one of the long edges of the PP release zone was bounded by parkland that blocked immigration and may help explain the slow but uninterrupted spread observed there (S2 Fig).

Seasonal variations in invasion dynamics are expected when mosquito abundance varies throughout the year. Models of *Wolbachia* population dynamics show that when mosquito host abundance fluctuates seasonally, *p* is expected to decline in the low seasons because of slow recruitment and comparatively high mortality among infected imagoes [57]. This was not observed in PP but was in EHW, where during D2 the area covered by the infection shrank and offsite *p* dropped considerably, only to recover the following wet season. PP may have been shielded from these effects by its apparent abundance of good mosquito habitat, reflected in its high trap yields throughout the study. Very few mosquitoes were caught in EHW during D2, and the sluggish recruitment there was a likely cause of the retraction of the invasion during that season.

Initial onsite infection frequencies at EHW correlated positively with window screens and negatively with habitat quality (S8 Table). This corroborates the findings of Hoffmann et al. [32] that following mass release of infected mosquitoes within an area, *Wolbachia* frequencies are highest in areas of poor mosquito habitat. However, no relationship was found between trap yields and simple measures of habitat quality (S2 Text). While BG-Sentinel traps can pick up on seasonal changes in mosquito abundance [58,59], they may not be able to give precise estimates of local *Ae*. *aegypti* densities at the scale of deployment used in this study.

Modelling offsite spread as a function of easily observed habitat variables was inconclusive (S2 Text). No variables were consistently predictive across seasons or sites, and in some cases variables that were expected to encourage spread (i.e., areas of low *Ae*. *aegypti* density: those with window screens, low-set dwellings, poor habitat quality) were found to deter it. However, predictions based on variation in population density and uniform dispersal (e.g., [20]) may be confounded by active searching for favourable oviposition sites [60], if density variation is driven by local habitat quality.

The lack of any discernible predictor variables, the strongly heterogeneous spread, and the drop in infection frequencies at the centre of the EHW release zone during W2 suggest that stochastic processes may have played a role in the invasions of EHW and PP. This is surprising considering that the scale of the Cairns invasions was much larger than those thought to be susceptible to stochastic effects associated with very small numbers of infected individuals [61]. Alternatively, a series of highly localised processes may have influenced the heterogeneity of the spread. If this is the case, our BG-Sentinel traps may be too dispersed to pick up on local variability that could inform future releases [62]. Spatial structure in *Ae*. *aegypti* populations has been observed at the house scale in Cairns [63]; in ensuing releases of *Wolbachia*-infected *Ae*. *aegypti*, a more clustered placement of traps within and around the advancing wavefront may provide a clearer picture of the processes at work. Despite the heterogeneity, our simple 2-parameter model seems to plausibly account for the slow rates of spread observed at our larger release sites. Bistability for the *w*Mel transinfection, versus the apparent tendency for successful natural *Wolbachia* infections to spread even when very rare, accounts for the fact that spread in Cairns is orders of magnitude slower than observed *Wolbachia* spread in natural *Drosophila* populations [30,31].

In summary, we have found rapid local establishment of *w*Mel *Wolbachia* in the *Ae*. *aegypti* populations of urban release areas, with an adjacent suitable habitat available for mosquito dispersal. In the 2 release areas that exceeded the predicted minimum size threshold for local establishment, the infection remained at a moderately high frequency for 2 years. Moreover, *w*Mel spread slowly outward at a rate consistent with theoretical predictions, based on realistic estimates of local dispersal and the position of the unstable equilibrium frequency. While this rate of *Wolbachia* spread is extremely slow, these findings indicate that large urban areas can be transformed gradually with patchy local *Wolbachia* releases [31]. Local information about barriers to dispersal can inform the minimum number of releases required, but it remains a challenge to understand the heterogeneity of spatial spread in terms of easily obtained data concerning habitat quality.

## Methods

### Study area

*Ae*. *aegypti* adults infected with the *w*Mel strain of *Wolbachia* were released by Eliminate Dengue staff at 3 zones in Cairns, Queensland, from January to April 2013. Overall, 131,420 mosquitoes were released at EHW, 286,379 at PP, and 35,196 at WC. More mosquitoes were released at PP because of its denser local *Ae*. *aegypti* population, and the 3 sites began with comparable proportions of wild and introduced mosquitoes.

The largest of the zones, EHW, was also the most open, situated amid residential suburban development with no potential dispersal barriers in its vicinity. PP and WC were in contrast both semiclosed, with each having 1 side of the release zone bounded by parkland so that from the centrum only 268° of the release zone at PP and 254° at WC was connected directly to urbanised *Ae*. *aegypti* habitat. Additionally, both PP and WC were near major roads, specifically Mulgrave Road to the southeast and Captain Cook Highway to the northeast (Fig 1), which could act as barriers to mosquito dispersal. These roads each consisted of 6–10 lanes totalling >50 m throughout. They were flanked by commercial buildings, for the most part, interspersed with apartment complexes.

Due in part to an abundance of modern single-storey housing, EHW was known to support a lower density of *Ae*. *aegypti* than the other sites. In contrast, PP was adjacent to Cairns’ central business district, and its household size of 2.00 per dwelling—smaller than that of either EHW (2.29) or WC (2.11) (http://profile.id.com.au/cairns/population)—reflects a larger number of multistorey apartment complexes, fewer bungalows and a higher density of unscreened older houses. At each of the 3 locations, the area enclosed by the release zone was thought to support a higher density of *Ae*. *aegypti* than the area surrounding the release zone, where there tended to be a higher density of modern houses. Cairns experiences a tropical monsoon climate, with a wet season running from November to April.

The successful establishment and spatial spread of *Wolbachia* following releases requires that the release area exceed a theoretical minimum, described by a critical radius *R*_crit_ [20,31]. While both EHW and PP clearly surpass the minimum, the small area of WC makes establishment there uncertain. Numerical analyses show that *R*_crit_ depends on both the shape of the dispersal function and the average dispersal distance, with small releases more likely to be successful with lower dispersal that is highly leptokurtic (i.e., showing both more long-distance and more short-distance dispersal that expected under a Gaussian function [31]). Dynamics of establishment and spread likewise depend on the size of the release zone relative to *R*_crit_, with faster dynamics predicted when the release zone is very large or very small relative to the minimum size. From this, we expect EHW and PP to display relatively rapid spread. In contrast, given that the release area at WC is close to the minimum, the failure or success of establishment is expected to take on the order of 2 years (about 20 generations) to ascertain [31].

### BG-Sentinel trap collections

BG-Sentinel mosquito traps (Biogents AG, Regensburg, Germany) were set up within and around each release zone at fixed positions in the yards of consenting householders, covering a distance of 25–530 m (EHW), 35–670 m (PP), and 30–520 m (WC) from the release zones in every available direction. The exact number of trapping sites varied over time as traps were moved from households whose residents had either moved or decided to terminate participation in the project. New traps were also added occasionally. By April 2015, data had been collected from 182 traps at EHW (44 onsite traps within the release zone, 138 offsite traps outside the release zone), 142 traps at PP (42 onsite, 100 offsite), and 74 traps at WC (20 onsite, 54 offsite). BG-Sentinel traps catch mosquito adults by means of a visual lure (black entry cup) and suction fan. In Cairns, they capture *Ae*. *aegypti* with high specificity and in large numbers [59].

Traps were checked weekly from 7 May 2013 to 30 April 2015. Traps that failed because of malfunction, invasion by predators (ants, spiders) or physical disturbance were scored as null observations for that time point. Adult mosquitoes from each trap were stored in ethanol at –20°C. Traps at WC ceased being checked after 1 April 2015, as new releases began in the area.

Samples were shipped to Monash University, where *Wolbachia* frequencies in mosquitoes from individual traps were determined by PCR using methods as previously described [14], with the following modifications. Samples were run through a multiplex qPCR assay with Taqman probes to detect *Wolbachia* and confirm identification of *Ae*. *aegypti* in the same reaction. Samples were extracted in 50-μL squash buffer (10 mM Tris pH 8.4, 1 mM EDTA, 50 mM NaCl) with 1.25% Proteinase K with a mini-beadbeater for 1.5 minutes and then incubated at 56°C for 5 minutes, incubated at 98°C for an additional 5 minutes, and then kept at 4°C until run. PCR reactions were run in a 10-μl total volume consisting of Lightcycler 480 mastermix, 1 μl of DNA extract, and primers and probes as follows. Species identification was determined with *Ae*. *aegypti* ribosomal protein gene RPS17 using Rps17_FW: 5′-TCCGTGGTATCTCCATCAAGCT-3′, Rps17_RV: 5′-CACTTCCGGCACGTAGTTGTC-3′, with Rps17_TaqM_Probe: 5′-**FAM**-CAGGAGGAGGAACGTGAGCGCAG-**BHQ1**-3′. *Wolbachia* infection status was determined with *w*Mel gene WD0513 using TM513_F: 5′-CAAATTGCTCTTGTCCTGTGG-3′, TM513_R: 5′-GGGTGTTAAGCAGAGTTACGG-3′, with TM513_TaqM_probe: 5′-**LC640**-TGAAATGGAAAAATTGGCGAGGTGTAGG-**Iowablack**-3′. Analysis was done by absolute quantification and the second derivative method in Roche Lightcycler software.

*Ae*. *aegypti* abundances are known to vary considerably throughout the year in Cairns [64]. This led us to partition the trap data into seasonal units, reflecting the 6-month wet and dry seasons of Northern Queensland. The first dry season D1 (May 2013–October 2013) began immediately after the releases, followed by the first wet season W1 (November 2013–April 2014), the second dry season D2 (May 2014–Oct 2014), and the second wet season W2 (Nov 2014–Apr 2015). This allowed comparisons across time as well as space.

For our graphical analyses, we aggregated data for each trap to give the following: (1) total mosquito abundance per season, (2) total number infected with *Wolbachia* per season, (3) an average number of mosquitoes observed per week (total and infected), and (4) a seasonal infection frequency, *p*. As in Hoffmann et al. [18], we checked species identity and *Wolbachia* infection status by PCR. For each mosquito, PCR was performed using 3 primer sets, *Aedes* universal primers (*mRpS6*_F/*mRpS6*_R), *Ae*. *aegypti*–specific primers (*aRpS6*_F/*aRpS6*_R), and *Wolbachia*-specific primers (*w1*_F/*w1*_R).

### Analyses of spatial spread: Mathematical background

#### Mathematical background

Before describing the graphical and statistical analyses, we review key theoretical results summarized in Barton and Turelli [20] and Turelli and Barton [31] concerning spatial spread with bistable dynamics. Let $\hat{p}$ denote the threshold frequency above which the *w*Mel infection frequency is expected to locally increase. A realistic model of *w*Mel frequency dynamics must minimally include overlapping generations with age structure [27] and density dependence [53,65]. Hence, $\hat{p}$ is best viewed as a convenient summary statistic indicating the relative fitness costs associated with *w*Mel in comparison to the frequency-dependent advantage produced by CI [27]. Turelli and Barton [31] summarize empirical evidence suggesting that near Cairns, $\hat{p}$ for *w*Mel in *Ae*. *aegypti* is likely to be in the range 0.25–0.35. Dispersal behaviour is summarized by the parameter *σ*, the standard deviation of dispersal distances per generation along any given axis. Assuming Gaussian dispersal, the mean Euclidean distance between the birthplaces of mothers and daughters is $\sigma \sqrt{\pi /2}\approx 1.25\sigma$.

As reviewed by Barton and Turelli [20], a partial differential equation model for spatial-temporal dynamics, with an idealized cubic approximation for local infection-frequency dynamics, provides analytical predictions for the rate of spatial spread and the shape of the spreading wave [66]. Assuming complete CI (i.e., all embryos produced from incompatible crosses die), the predicted wave speed is

$$c=\sigma (\frac{1}{2}-\hat{p})$$ (2)

per generation. The spreading wave assumes a characteristic asymptotic shape. If we define wave width, *w*, as the inverse of the maximum slope of infection frequency [49], the cubic-diffusion approximation produces

w=1/Max(|∂p/∂x|)=4σ. (3)

Prediction 3 provides an estimate of average dispersal distance from spatial infection frequency data once steady spatial spread is initiated. Using the asymptotic wave formula that generates Eqs 2 and 3, we expect infection frequencies to change from about 0.18 to 0.82 over 3σ. (Similarly, infection frequencies are expected to change from 0.3 to 0.8 over about 2.23σ.) Using estimates of *c* and *σ* with Eq 1, we can approximate $\hat{p}$ from joint estimates of speed and width––assuming these analytical predictions are robust.

Turelli and Barton [31] examined the robustness of predictions 2 and 3 to both long-tailed dispersal and the rapid local changes in infection frequency expected with complete CI. Relation 2 between wave width and *σ* is quite robust, with maximum departures on the order of 10% for $0.2\leq \hat{p}\leq 0.35$. In contrast, over the same range of parameters and models, long-tailed dispersal (corresponding to higher frequencies of both long-distance and short-distance dispersal than expected under a Gaussian) with complete CI can reduce wave speed by 10%–40%. Hence, estimates of σ from observed wave width and prediction 3 are likely to be quite robust, whereas wave speed may be systematically overestimated by prediction 2.

Barton and Turelli [20] presented conditions for wave initiation and wave stopping. To avoid being swamped by immigration of uninfected individuals, releases must cover a sufficiently large area to initiate an expanding wave. Assuming that $\hat{p}\leq 0.35$, releases within circles of a radius greater than 3σ should suffice to initiate spatial spread. As shown by Turelli and Barton [31], even smaller releases should initiate spread with long-tailed dispersal, especially if *Wolbachia*-induced fitness reductions mainly involve fecundity. However, bistable waves can be relatively easily stopped by environmental heterogeneities and barriers to dispersal such as roads. This phenomenon is illustrated by the *w*Mel frequency data from Pyramid Estate, a small suburb separated by a highway from the 2011 release site in Gordonvale, about 20 km south of central Cairns. As reported by Hoffmann et al. [18], the *w*Mel infection frequency has remained stable at over 95% in Gordonvale since 2011. Occasional *w*Mel-infected *Ae*. *aegypti* migrate across the highway to Pyramid Estate. Yet, the infection frequency has never increased appreciably in Pyramid Estate and the long-term average is only 0.106. As noted by Turelli and Barton [31], this provides an approximate lower bound of $\hat{p}\geq 0.21$.

Bistable waves can be slowed or stopped by increases in population density. In general, increases in population density slow wave speed. However, as $\hat{p}$ approaches 0.5, even very small increases suffice to stop wave spread [20]. Hence, natural heterogeneity in *Ae*. *aegypti* population density is likely to produce heterogeneous rates of spatial spread.

### Analyses of spatial spread: Graphical analyses of infection-frequency data

#### Graphical analyses of infection-frequency data

Ordinary Kriging [67] was performed to interpolate data on a map and visualize the patterns of spread based on the seasonal *p* of traps from which at least 4 mosquitoes had been collected in the season. Interpolative maps were created predicting *Wolbachia* frequencies throughout each site, from which the direction and extent of the invasion could be inferred. Kriging was performed in ArcMap 10.2.2 [52] using an exponential semivariogram model and a 24-point nearest-neighbour search function for EHW and PP and a 16-point nearest-neighbour search function for WC (because of fewer traps at WC).

Kriging maps of infection frequencies averaged over 6-month windows allowed for the rough approximation of *c* and *σ*. To approximate *c*, we first calculated the areas enclosed by the *p* ≥ 0.5 and *p* ≥ 0.8 map contours for each season, using ArcMap 10.2.2. These contours defined areas within which *Wolbachia* frequency was greater than the highest possible estimates of $\hat{p}$ consistent with spatial spread (roughly $\hat{p}\geq 0.5$, [20]) and areas within which *Wolbachia* was near fixation. With each plot representing 6 months of spread, the increase in area can be used to estimate wave speed using formulas provided below. To estimate *σ*, we calculated for each season the average distance between the *p* ≥ 0.3 and *p* ≥ 0.8 map contours. This was achieved by plotting 36 lines at 10° intervals from the centre of each of the EHW and PP release zones. From where each of these lines intersected the *p* ≥ 0.8 contour, we calculated the shortest distance to the *p* ≥ 0.3 contour.

To translate the estimates of expanding *Wolbachia*-infected areas into heuristic approximations of wave speed, we used simple geometric models. Consistent with our likelihood analyses below, we assume that 6 months after the releases began, the infection approached its asymptotic rate of spatial spread, *c*. The relationship between changes in area and speed of the wave front, *c*, depends on the shape of the release area and whether spread can occur in all directions. The closed curve of fixed length that encloses the largest area is a circle, hence we can approximate the maximum rate of uniform spread in all directions from a release by assuming a circular release area with symmetric spread in all directions. If the area in which the infection has frequency ≥ 0.5 increases from *A*_0_ to *A*_*t*_ over *t* generations, the wave speed per generation is

$$c=\left(\sqrt{A_{t}}-\sqrt{A_{0}}\right)/\left(t\sqrt{\pi }\right).$$ (4)

To examine the effect of release-area shape, we can instead assume that the release occurs in a rectangle, with the long sides *y* times longer than the short (so *y* = 1 with a square release area). As the infection spreads, the area covered has quarter-circle corners, asymptotically approaching a circle through time. With a rectangular release, the relationship between area change and wave speed is

$$c=\left(\sqrt{{\left((1+y)\sqrt{A_{0}/y}\right)}^{2}+\pi (A_{t}-A_{0})}-(1+y)\sqrt{A_{0}/y}\right)/\left(t\sqrt{\pi }\right).$$ (5)

Finally, suppose that the infection can spread in only 3 directions, with no expansion possible in the direction of one of the longer sides (so that the expanding wave asymptotically becomes a semicircle). Then wave speed can be approximated as

$$c=\left(\sqrt{{\left((2+y)\sqrt{A_{0}/y}\right)}^{2}+2\pi (A_{t}-A_{0})}-(2+y)\sqrt{A_{0}/y}\right)/\left(t\sqrt{\pi }\right).$$ (6)

We apply these approximations to the EHW and PP data below and compare them to model-based likelihood estimates. Note that *c* and *t* can be measured in generations or in days.

#### Likelihood analyses

To estimate wave speed and wave width, we reduce the two-dimensional data to one dimension by calculating distances from the edge of the release area and averaging over time and space.

*Defining time and distance*: Days are counted from 3 October 2012; the first collection at EHW and PP was on day 104 (15 January 2013), and the last collections were on days 882, 881 (4, 3 March 2015) respectively. Releases began in 10 January 2013 (day 99) and ended on 18 April 2013 (day 197). The data were reduced to one dimension by measuring the distance r* from the nearest edge of the release area, with negative values assigned to points inside the release areas. For EHW (PP), the sample point within the release area that was farthest from the edge had distance *r*_min_ = −340.1 m (−220.2 m). The mean position of the vertices of the release area was defined as {x_0_, y_0_}. At EHW (PP), {x_0_, y_0_} was a distance −351.1 m (−155.5 m) from the nearest edge. The effective radial distance was taken to be *r* = r* − *r*_min_, so that the sample point within the release area farthest from the release area edge has *r* = 0.

*Statistical model for estimating wave speed and width*: Instead of fitting a mechanistic model of temporal and spatial dynamics, we fit a statistical model that approximates both initial conditions after our releases and the spreading wave. The model assumes the spreading wave shape approximates the one-dimensional asymptotic solution of the cubic diffusion approximation (see Eq. 13 of [20]). For each time, we approximate the spatial distribution of infection frequencies as a function of distance from the release area, *r*, using

$$p(r)=1/\left[1+\frac{2\pi \nu }{\alpha }Exp\left(\frac{r^{2}}{2\nu }\right)\right],$$ (7a)

where

$$\alpha =\left(\frac{\pi wr_{0}}{2}\right)Exp\left(\frac{2r_{0}}{w}\right)\text{ and }\nu =\frac{wr_{0}}{4}.$$ (7b)

**Fig 8:**
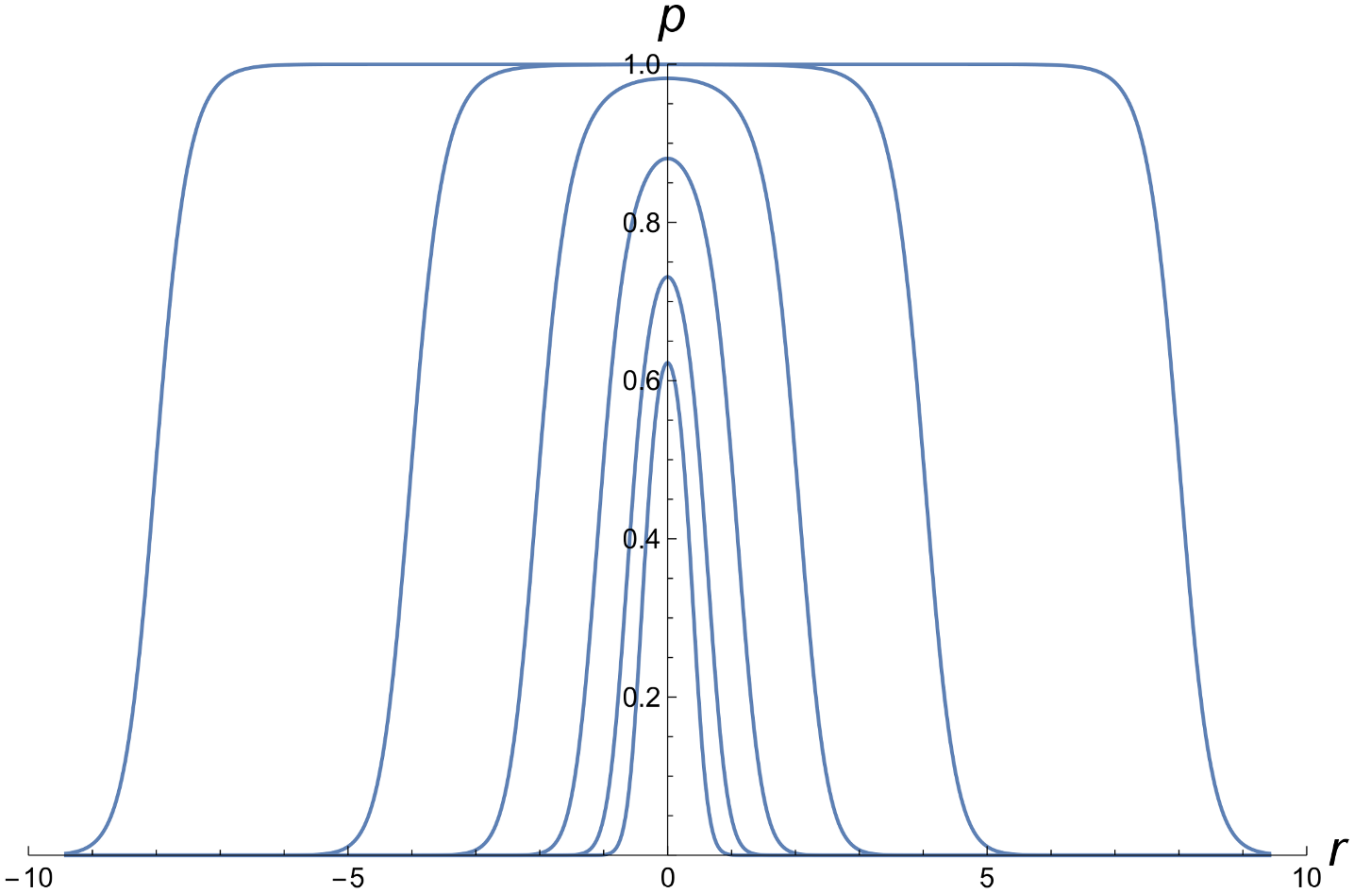
The statistical Model (7) describing the position and width of the wave. Assuming wave width *w* = 1, the figure illustrates the transition from a near-Gaussian distribution of infection frequencies near the centre of the release (for *r*_0_ = 0.25, 0.5) to a wave traveling in both directions (for *r*_0_ = 1, 2, 4, 8).

In *p*(*r*), *r*_0_ indicates the position of the wave when *r*_0_ > *w*. For small *r*_0_ (*r*_0_ << *w*), Model 7 is approximated by a Gaussian distribution scaled to total mass *α*, i.e.,

$$p(r)\approx \frac{\alpha }{\sqrt{2\pi \nu }}Exp\left(-\frac{r^{2}}{2\pi \nu }\right),$$ (8)

where *α* and *v* are given by Eq (7b). For large *r*_0_, Model 7 approaches a logistic cline with width *w* centred at *r*_0_, i.e.,

$$p(r)=1/\left[1+Exp\left(\frac{4(r-r_{0})}{w}\right)\right],$$ (9)

where *w* is cline width, as defined in Eq 2. Fig 8 shows *p*(*r*) from Model 7 for various *r*_0_ with *w* = 1.

#### Pooling data

As a first approximation, data were pooled into 9 time intervals with boundaries at 90, 110, 120, 150, 250, 300, 400, 550, 700, and 900 days and distances pooled at 100-m intervals, counting relative to the edge of the release area (r* = 0). Maximum likelihood estimates of the parameters *r*_0_ and *w* of the Gaussian/logistic Model (7) were obtained separately for each time interval, initially assuming that binomial sampling is the only source of variation in frequencies at each distance from the release boundary. Other sources of variation are considered below.

To estimate wave speed, we calculated the slope of the regression of *r*_0_ against time once rough linearity was achieved. Similarly, the wave width was estimated as the average value of *w* once spread had become roughly linear (see Results).

In S1 Text, we describe 3 additional likelihood analyses. The first provides a simple model to account for variance in infection frequencies above that expected from binomial sampling alone. The second describes an analysis of wave speed and width without pooling the data over time or space. The third is an explicit analysis of the spatially heterogeneous spread at EHW, which supports the heterogeneity apparent from Fig 3.

## Supporting information

S1 Fig Onsite infection frequency (*p*) at EHW and PP from the onset of releases to the end of D1. The white circle marks the end of releases on 18 April 2013. Infection frequencies remained stable after releases ended.
(TIFF)

S2 Fig Area enclosed by the *p* > 0.8 and *p* > 0.5 contours. The area enclosed by the *p* > 0.8 and *p* > 0.5 kriging contours was calculated for each season at EHW (A), PP (B) and WC (C). The area covered by the infection at EHW and PP tended to increase over time, while at WC it decreased following a high at W1.
(TIFF)

S3 Fig Increasing *Wolbachia* infection frequencies through space and time. Panels A and C show the pooled data from EHW and PP; panels B and D show the maximum likelihood fits of those data to the model described by Eq (7). The *x*-axis in each panel is distance (in meters) from the edge of the release area, the *y*-axis is infection frequency. Each of the nine colored lines shows the spatially spreading infection, the first centred on *t* = day 100 (releases began on day 99, blue), the last centered on day 800, dark red), nearly two years after the releases were completed on day 197. The midpoints of the intermediate time intervals are at 100 (blue), 115, 135, 200 (green), 275, 350 (yellow), 475, 625 and 800 (dark red) days. Note that only eight curves appear in Panel D, because the curves generated by the data from the second and third time interval are coincident.
(TIFF)

S4 Fig Heterogeneous spatial spread at EHW. The picture shows estimated contours of *w*Mel frequency surrounding the EHW release area, together with averages after day 800. The average sampling date is day 847, approximately 750 days after the final releases. The contours correspond to frequencies of 0.9, 0.7, 0.5, 0.3, 0.1 (starting from the center). The 50% contour is thickest. The picture shows the best-fitting model in each four equal-angle sectors centered on the EHW release area. The release area is in pale blue, the red dots are the release points. The area of each pie is proportional to sample size, and the shaded portion is proportional to infection frequency.
(TIFF)

S5 Fig Offsite infection frequency (*p*) at the three study sites. The average infection frequency among mosquitoes caught in offsite traps is shown for each season at each site. At PP, *p* increased almost linearly. At EHW, *p* was considerably more volatile, though it showed a clear tendency to increase with time. At WC, *p* decreased slowly after D1.
(TIFF)

S1 Table Number of *Ae*.*aegypti* caught in traps. Total and infected *Ae*. *aegypti* within (onsite) and adjacent to (offsite) each release area.
(XLSX)

S2 Table Likelihood analyses of wave position and width for EHW and PP using a binomial model. Likelihood analyses of infection frequencies using data pooled over time and by distance from release sites. These analyses account for only binomial sampling variance. For each time interval, the pooled data are used to estimate the parameters *r*_0_, describing the position of the wave, and *w*, the wave width, in Eq (7).
(XLSX)

S3 Table Likelihood analyses of wave position and width for EHW allowing for non-binomial sources of variation. EHW estimates of the parameters in Model (7) obtained with model (S1) to account for non-binomial sources of variation in infection frequencies via the parameter *F*. For each time interval, we provide Log(*L*) and the MLEs for *r*_0_ and *w* with interval-specific *F* (left), or with a common *F* = 0.226 (right).
(XLSX)

S4 Table Likelihood analyses of wave position and width for PP allowing for non-binomial sources of variation. PP estimates of the parameters in Model (7) obtained with model (S1) to account for non-binomial sources of variation in infection frequencies via the parameter *F*. For each time interval, we provide Log(*L*) and the MLEs for *r*_0_ and *w* with interval-specific *F* (left), or with a common *F* = 0.167 (right).
(XLSX)

S5 Table Heterogeneity of spatial spread at EHW. Estimates of wave speed per day (*c*_d_) and wave width (*w*) in four directions. The four rows correspond roughly to south, east, north and west in Fig 3.
(XLSX)

S6 Table Likelihood analyses of wave position and width for WC allowing for non-binomial sources of variation. WC estimates of the parameters in Model (7) obtained with model (S1) to account for non-binomial sources of variation in infection frequencies via the parameter *F*. For each time interval, we provide Log(*L*) and the MLEs for *r*_0_ and *w* with interval-specific *F* (left), or with a common *F* = 0.1 (right).
(XLSX)

S7 Table Moran's *I* index values showing spatial clustering across sites and seasons. *I* was calculated as a measure of spatial autocorrelation in trap yields of all *Ae*. *aegypti*, all infected *Ae*. *aegypti*, and all uninfected *Ae*. *aegypti*, and in trap infection frequencies. Shaded cells indicate statistical significance at *P* < 0.05.
(XLSX)

S8 Table Statistical analyses of effect of housing variables using GLMM. Housing variables (i) and habitat quality (ii) were averaged over 100 m radii. Shaded cells indicate statistical significance at *P* < 0.05. Distance units are in metres, housing variables are scored between 0 and 1, and habitat quality is scored between 0 and 3. Coefficients are presented in logit scale. Distance from the release zone (distance) was the only robust predictor of the infection.
(XLSX)

S1 Text Supporting Information concerning additional likelihood analyses and results. (DOCX)

S2 Text Supporting information concerning observed *w*Mel frequencies and analyses of habitat variables. (DOCX)

S1 Data Excel file with data on mosquito densities, *Wolbachia* infection status and housing characteristics. (XLSX)

S2 Data Text file describing the data format in S1 Data. (TXT)

## Abbreviations

CI: cytoplasmic incompatibility
D1: first dry season
D2: second dry season
EHW: Edge Hill/Whitfield
MLE: maximum likelihood estimate
PP: Parramatta Park
W1: first wet season
W2: second wet season
WC: Westcourt
