# Supplementary figures and images for: Local introduction and heterogeneous spatial spread of dengue-suppressing Wolbachia through an urban population of Aedes aegypti
Source: PLoS Biol. 2017 May 30;15(5):e2001894. doi: 10.1371/journal.pbio.2001894 (PMC5448718; doi:10.1371/journal.pbio.2001894)

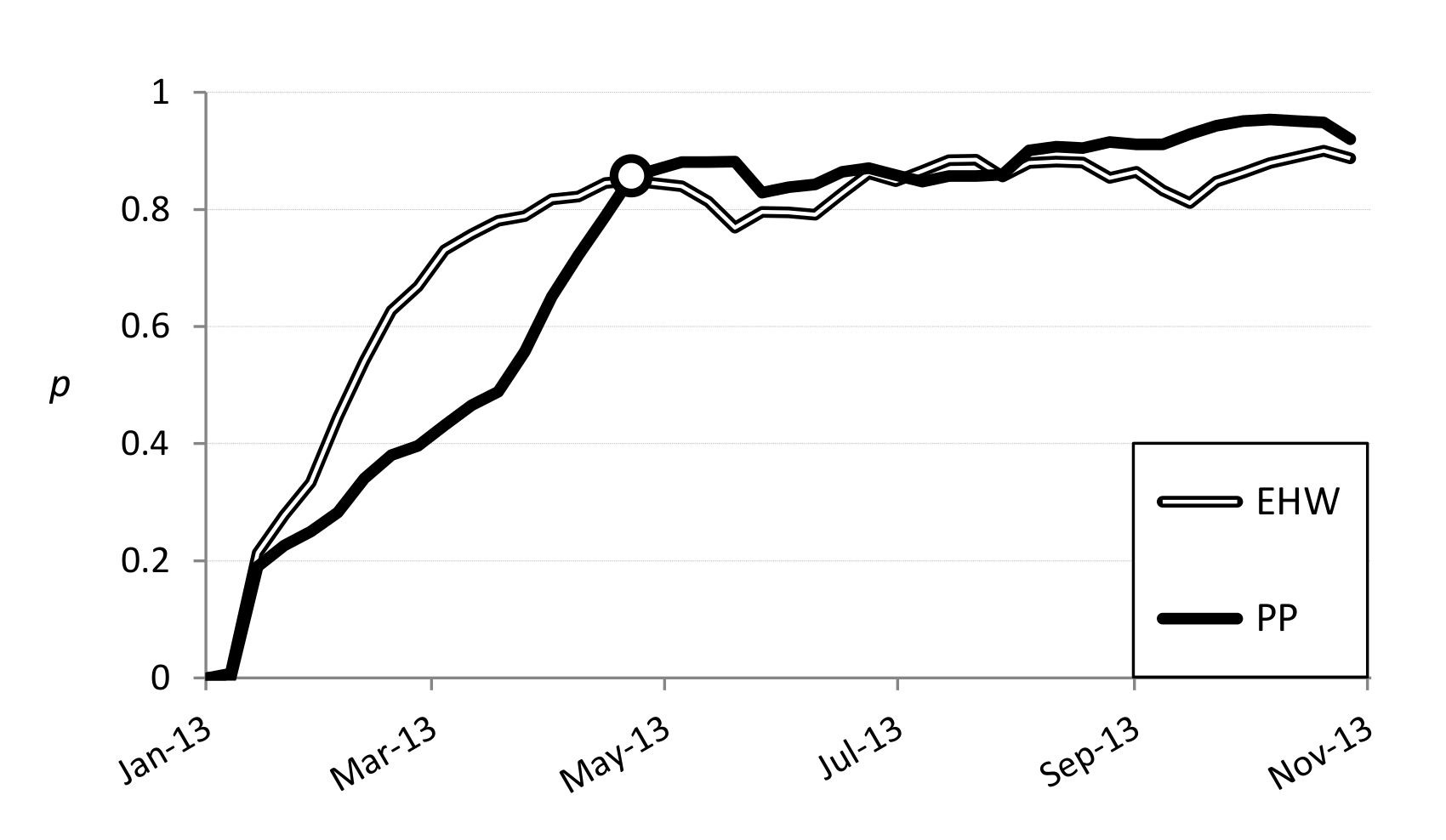

Supplement: S1 Fig — The white circle marks the end of releases on 18 April 2013. Infection frequencies remained stable after releases ended. (TIFF) [file pbio.2001894.s001.tiff]

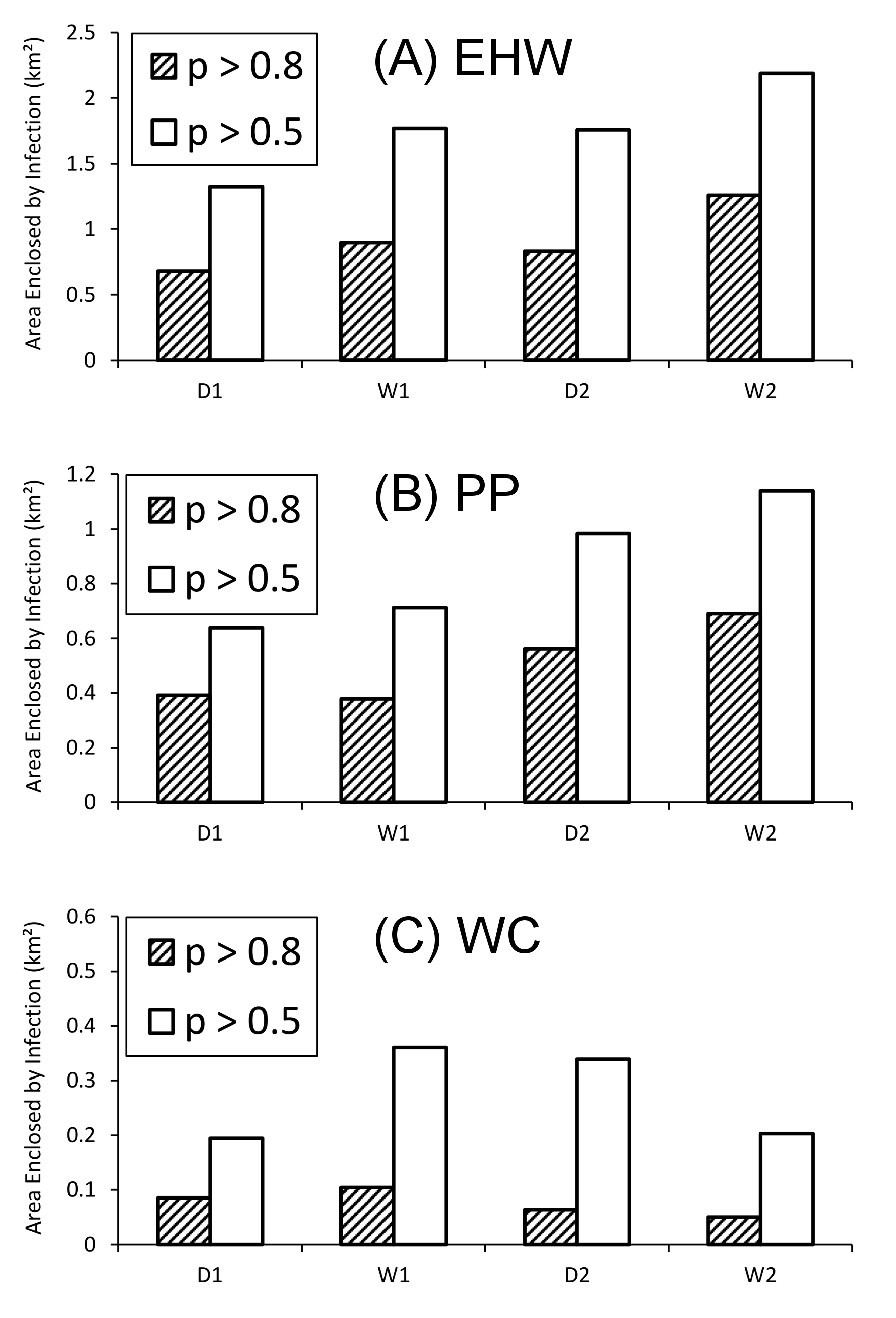

Supplement: S2 Fig — The area enclosed by the p > 0.8 and p > 0.5 kriging contours was calculated for each season at EHW (A), PP (B) and WC (C). The area covered by the infection at EHW and PP tended to increase over time, while at WC it decreased following a high at W1. (TIFF) [file pbio.2001894.s002.tiff]

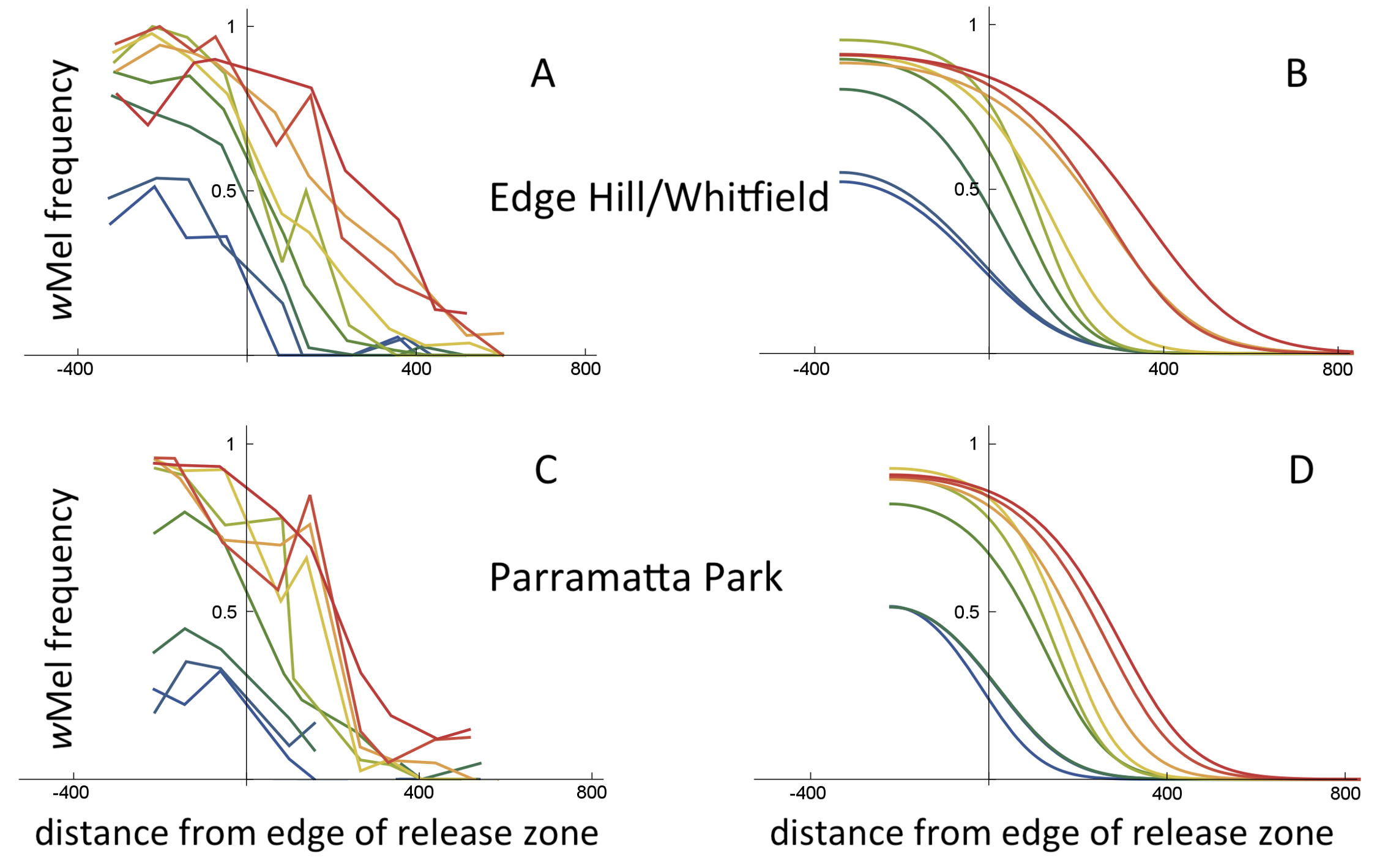

Supplement: S3 Fig — Panels A and C show the pooled data from EHW and PP; panels B and D show the maximum likelihood fits of those data to the model described by Eq (7). The x-axis in each panel is distance (in meters) from the edge of the release area, the y-axis is infection frequency. Each of the nine colored lines shows the spatially spreading infection, the first centred on t = day 100 (releases began on day 99, blue), the last centered on day 800, dark red), nearly two years after the releases were completed on day 197. The midpoints of the intermediate time intervals are at 100 (blue), 115, 135, 200 (green), 275, 350 (yellow), 475, 625 and 800 (dark red) days. Note that only eight curves appear in Panel D, because the curves generated by the data from the second and third time interval are coincident. (TIFF) [file pbio.2001894.s003.tiff]

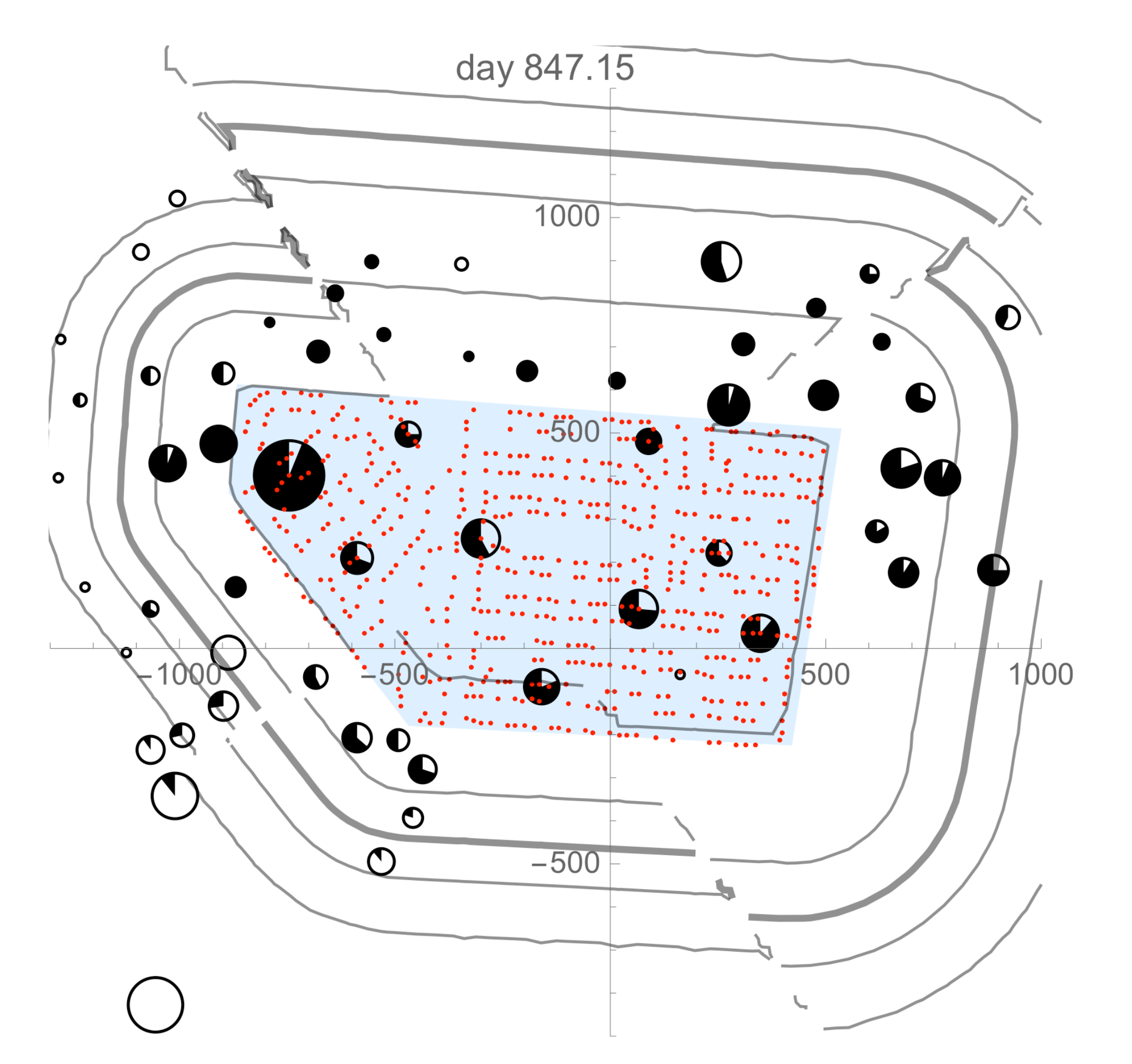

Supplement: S4 Fig — The picture shows estimated contours of wMel frequency surrounding the EHW release area, together with averages after day 800. The average sampling date is day 847, approximately 750 days after the final releases. The contours correspond to frequencies of 0.9, 0.7, 0.5, 0.3, 0.1 (starting from the center). The 50% contour is thickest. The picture shows the best-fitting model in each four equal-angle sectors centered on the EHW release area. The release area is in pale blue, the red dots are the release points. The area of each pie is proportional to sample size, and the shaded portion is proportional to infection frequency. (TIFF) [file pbio.2001894.s004.tiff]

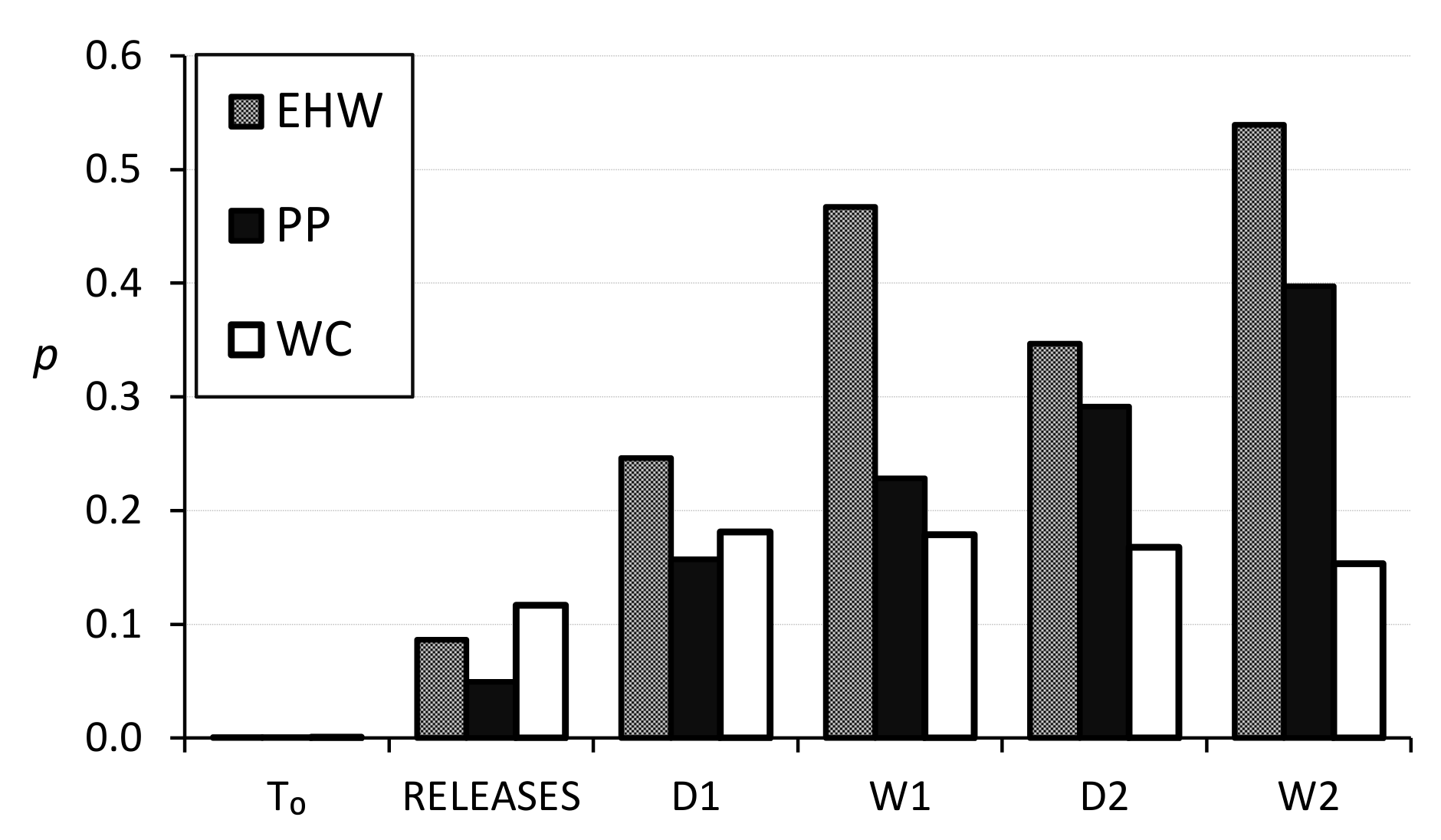

Supplement: S5 Fig — The average infection frequency among mosquitoes caught in offsite traps is shown for each season at each site. At PP, p increased almost linearly. At EHW, p was considerably more volatile, though it showed a clear tendency to increase with time. At WC, p decreased slowly after D1. (TIFF) [file pbio.2001894.s005.tiff]
