# Supplementary material for: Local introduction and heterogeneous spatial spread of dengue-suppressing Wolbachia through an urban population of Aedes aegypti
Source: PLoS Biol. 2017 May 30;15(5):e2001894. doi: 10.1371/journal.pbio.2001894 (PMC5448718; doi:10.1371/journal.pbio.2001894)
Supplement: S1 Text — (DOCX) [file pbio.2001894.s014.docx]

**S1 Text**. **Supporting Information concerning additional likelihood analyses and results**

**1.1 Accounting for excess variance above binomial sampling**

*1.1.1. Methods: A simple model accounting for additional variance*

The estimated infection frequencies at a given distance from the release areas vary more than can be explained by binomial sampling alone. Finite population size inevitably generates excess variance, and additional sources of variance associated with habitat heterogeneity also influence local infection frequencies (e.g., we expect areas of higher population density to be invaded more slowly). Our one-dimensional approximation (7) pools data over such heterogeneities. To account for additional sources of variation, we assume that the underlying infection frequency at each distance follows a beta distribution with mean *p* and variance *Fp*(1 – *p*). Under this model, the probability of finding *i* infected individuals in a sample of size *n* is

(S1a)

where and . (S1b)

As *F* → 0, the probability model converges to a binomial with parameters *n* and *p*. For a single individual (*n* = 1), infection probability is simply *p*, as expected. With larger *n*, the effect of allowing for *F* > 0 is roughly to reduce the weight given to large samples. The parameters *r*_0_ and *w* in model (7) were estimated both with and without accounting for this additional variation.

*1.1.1. Results*

We re-estimated the parameters *r*_0_ and *w*, describing wave position and wave width, using the sampling model (S1), which introduces the parameter *F* describing additional sources of variation. Two approaches were taken: we estimated *F* separately for each of the nine time intervals (see S2Table) and alternatively estimated a single *F* assumed to apply across all time intervals. The likelihood results are in S3 Table and S4 Table.

For EHW (S3 Table), when estimating a single *F*, we obtain the maximum likelihood estimate (MLE) *F* = 0.226. Estimating *F* separately for each time interval significantly improves the fit to the data, increasing the log likelihood by 18.93, which is highly significant (2ΔLog(*L*) is, *P* < 10^–4^). The standard deviation (sd) of the estimates of *F* is 0.11 (with range 0.077-0.363). Estimating *F* for each interval produces *c*_d_ = 0.469 m/day and *w* = 456 m. In contrast, assuming a constant *F* across intervals (*F* = 0.226), produces *c*_d_ = 0.477 m/day and *w* = 454 m. (Like our estimates from the pooled data, these were based on data after day 120). For simplicity we assume *F* = 0.226 in our subsequent EHW analyses.

For PP (S4 Table), if we assume a constant *F* while analysing the pooled data, we obtain the MLE *F* = 0.167. Assuming that *F* does not vary reduces the likelihood by 20.8 relative to allowing *F* to vary across the nine intervals (, *P* < 10^–5^ ). The sd of our estimates of *F* is 0.09 (with range 0.076-0.361). With varying *F*, we obtain *c*_d_ = 0.287 m/day and *w* = 384 m. Assuming a constant *F* = 0.167, we obtain essentially identical results *c*_d_ = 0.287 m/day and *w* = 390 m. (These estimates were based on data after day 150, as in our pooled analysis without *F*, S2 Table). For simplicity we assume *F* = 0.167 in our subsequent PP analyses.

**1.2. Likelihood analyses without pooling**

*1.2.1. Methods*

As shown in Fig 6 panels A and C, the EHW and PP data fit our expectations of linear asymptotic spread reasonably well. To more carefully analyse these data, we reanalyzed them without pooling over time or space to fit the model *r*_0_ = *R* + *c*_d_*t*, where *R* is a constant describing the distance from the center of each release area at which approximately linear spread was observed and *c*_d_ is the wave speed measured as m/day.

*1.2.1. Results: Likelihood fits without pooling data over space or time*

For EHW, after day 120, *r*_0_ increased approximately linearly with time. Discarding samples before day 120 and using *F* = 0.226, obtained in Section 1.1, the MLEs are: *R* = 282 m, *c*_d_ = 0.505 m/day, and *w* = 469 m. (Note that the edge of the EHW release area corresponds to roughly *r*_0_ = 340 m.) To calculate support intervals for each parameter, we hold that parameter fixed, and maximise likelihood with respect to the other two. The support intervals are the parameter values that are two units of log likelihood below the maximum; asymptotically, this corresponds to 95% confidence intervals. For PP, *r*_0_ increased approximately linearly with time after day 150. Discarding samples taken before day 150 and using the excess-variance parameter, *F* = 0.167, obtained above, the MLEs are: *R* = 252 m, *c*_d_ = 0.301 m/day, and *w* = 384 m. (Note that the edge of the PP release area corresponds to roughly *r*_0_ = 220 m.) Table 1 in the text summarizes these likelihood estimates and support intervals as well as our previous likelihood estimates, based on pooling, for *c*_d_ and *w*.

**1.3. Likelihood analysis of heterogeneous spatial spread**

*1.3.1. Methods*

At EHW, we analysed the rate of spread in different directions to statistically evaluate the heterogeneous spread apparent from the graphical analyses in Fig 3. To examine directional heterogeneity, we divided the samples into four sectors defined by the angle, θ, from a point at the mean of the vertices of the release area. East corresponds to θ = 0, and the angle is counted counter-clockwise, so that north corresponds to θ = π/2. To best fit the data, we introduce a parameter φ and define four sectors by the intervals (φ – π,  φ – π/2),  (φ – π/2,  φ ), (φ,  φ + π/2) and (φ + π/2,  φ + π). We varied φ from 0 to π/4 in increments of π/16. For each φ, we fit *R*, *c*_d_ and *w* separately for each sector.

*1.3.2. Results*

The highest likelihood is achieved with φ = 3π/16, which gives a highly significant increase in log likelihood of 128.57, for 10 degrees of freedom, in comparison to the best fit of the data using single (direction-independent) values of *R*, *c*_d_ and *w*. The MLEs are given in S5 Table. S4 Fig shows the estimated contours of *w*Mel frequency in each of the four sectors along with the average frequency data, approximately 750 days after the final releases. As expected from Fig 3, *w*Mel spread is fastest to the east and north and significantly slower to the south and west.
