# Supplementary material for: Local introduction and heterogeneous spatial spread of dengue-suppressing Wolbachia through an urban population of Aedes aegypti
Source: PLoS Biol. 2017 May 30;15(5):e2001894. doi: 10.1371/journal.pbio.2001894 (PMC5448718; doi:10.1371/journal.pbio.2001894)
Supplement: S2 Text — (DOCX) [file pbio.2001894.s015.docx]

**S2 Text**. **Supporting information concerning observed *w*Mel frequencies and analyses of habitat variables**

**2.1 Offsite infection frequencies**

Infection frequencies among mosquitoes caught in offsite traps at EHW and PP tended to increase over time (S5 Fig). At PP, the average *p* across all offsite traps increased linearly with each season at a rate of 0.08 per 6 month period. Average offsite *p* was more volatile at EHW, increasing sharply from D1 to W1, dropping sharply in D2, then rising again in W2, with an average seasonal increase of 0.10. It was unclear why *p* decreased during D2, but as trap yields were very low – only 145 mosquitoes were caught during the entire period – the decrease may be stochastic. In comparison to the offsite drop in *p*, onsite *p* increased by 0.07 in D2, to an unprecedented high of 0.96. Our likelihood analysis below indicates a steady, but spatially heterogeneous, spread of *w*Mel at EHW.

At WC, offsite *p* increased from the start of releases to D1, after which it underwent a slow decline of about 0.01 per season, finishing at 0.15 in W2. This, along with the fluctuating infection frequencies onsite, suggests an invasion undergoing a slow collapse, a conclusion supported by our likelihood analysis (see Fig 7).

**2.2 Spatial Dependence**

*2.2.1. Methods*

Spatial autocorrelation of trap data was investigated using Moran’s *I*, calculated from inverse distance relationships between traps. At each site and for each season, *I* was calculated for: 1) infection frequency (*p*), 2) number of infected mosquitoes collected, 3) number uninfected mosquitoes collected, and 4) number of mosquitoes collected in total. All calculations were run in ArcMap 10.2.2 [1].

*2.2.2. Results*

Infection frequencies among offsite traps exhibited strong spatial clustering at EHW and PP for all four seasons (S7 Table). At PP, the number of infected mosquitoes collected was spatially clustered across all seasons; at EHW, clustering was observed from season W1 onwards. Spatial clustering of both uninfected and total mosquito yields occurred in seasons W1, D2 and W2 at EHW and in D2 and W2 at PP. This persistent increase in spatial dependence of uninfected and total mosquito yields may reflect the successful invasion of uninfected demes surrounding the central release zone, which are expected to undergo decreases in population size due to CI.

In contrast, while infection frequencies at WC were spatially correlated across all seasons, by the end of W2 neither the infected nor total mosquito yields displayed significant spatial structure. Additionally, all estimates of Moran’s *I* for WC were lower at W2 than at D1, showing a disintegration of spatial structuring with the progression of the invasion. At EHW and PP, Moran’s *I* scores for every condition increased over this same time period.

**2.3 Spatial Relationships**

*2.3.1. Methods: Regression Modelling*

Generalised Linear Mixed Models (GLMMs) were produced to investigate the relationship between trap yields, infection frequency (*p*), and mosquito habitat. Considering that areas of lower mosquito density are expected to be invaded more quickly [2], we were interested specifically in whether variables representing habitat quality were predictive of *p*, and thus could act as a proxy for unknown mosquito densities in future release scenarios. We also investigated whether BS-Sentinel trap yields could be predicted by habitat variables. While the average spacing between traps in this study, of 118 ± 41m at EHW and 96 ± 52m at PP in D1, is of an order similar to that at which fine-scale spatial structure of trap yields has been observed (130 m [3]), structuring at finer scales has been recorded [4]. It would thus be possible for habitat to be representative of density when trap yields were not, if significant structure existed below the scale of trapping but at or above the house scale at which habitat variables were scored.

We chose three variables to represent habitat: building height, construction material/s and presence of window screens, and scored 3177 dwellings at EHW and 2388 at PP for each of them. We scored dwellings both within the release zones and in surrounding areas. The characteristics scored were chosen because they were simple and inexpensive to measure, and they could be observed without disturbing the homeowner. Unlike data concerning human occupancy, which may be more predictive of local *Ae. aegypti* densities, our variables could be easily assessed for every dwelling over large areas.

Building heights was scored from 0 (greater than two storeys, or two storeys and an open ground floor) to 0.5 (two storeys, built into the ground) to 1 (low set, built in to ground). It was assumed that buildings scoring lower on this scale would provide more shade for mosquitoes, particularly the large elevated “Queenslander” timber houses prevalent in the region, which frequently have an underfloor space suitable for mosquito habitation and lack window screening. Construction material/s were scored based on the presumed access they would give mosquitoes to the building, from 0 (wood only, cement sheeting, sheet metal) to 0.5 (brick and wood) to 1 (brick). Window screens were scored as 0 (absent) or 1 (present). The main difference between EHW and PP for these characteristics was in building height, EHW had many low set dwellings and PP more elevated Queeslander houses.

It was conjectured that the three variables together might better represent mosquito numbers than each individual variable. We computed a composite variable whereby each dwelling was scored from 0 (high set, wood/cement-sheeting and unscreened: best possible habitat) to 3 (low set, brick, screened: worst possible habitat), giving each variable equal weight. On average, dwellings at PP provided better mosquito habitat than those at EHW.

As the lot sizes associated with individual dwellings were generally smaller than the presumed typical dispersal distance of *Ae. aegypti* (i.e., 50-100 m), aggregated dwelling characteristics were used in statistical models for spread. From each trap location, house data were aggregated within a buffer of 100m radius to give average scores for each covariate and for habitat quality. Aggregating the data this way provided a better estimation of local conditions and lessened the influence of traps set on regionally uncharacteristic properties.

We constructed two series of GLMMs, each with EHW and PP considered as separate treatments. For the first we modelled habitat as a predictor of *p* and trap yields onsite in D1, immediately after releases, thus attempting to corroborate previous findings of faster onsite invasion in areas of presumed worse habitat [5]. For the second, we modelled habitat for all seasons as a predictor of *p* and trap yields among offsite traps. This would determine whether habitat could be predictive of spread through areas surrounding release zones following releases. Infection frequency was treated as a binary variable, scoring a number of ‘successes’ (infected mosquitoes) against a number of ‘trials’ (number of mosquitoes collected), and was transformed via a logit link function. For the second series of GLMMs we also included distance from the edge of the release zone as a continuous variable, as well as first order interactions between it and other predictors. For each GLMM, habitat was modelled both as three separate variables or the composite variable representing habitat quality.

GLMMs were run in R using the spaMM package [6] and fit using maximum likelihood. Spatial dependence in *p* was accounted for with the Matérn covariance function [7], with the Euclidean distances between traps used to calculate covariances. In all GLMMs, the Matérn correlation parameters ‘p’ and ‘v’ (scale and smoothness, respectively) were estimated from the data. Nonsignificant elements (*P* > 0.05) were removed from each model sequentially in order of nonsignificance, with first interactions removed before main effects.

*2.3.2. Results of Regression Modelling*

For the GLMMs modelling onsite variables, we found no evidence that habitat was predictive of D1 trap yields at EHW and PP. However, local abundance of window screens (coefficient: 2.511) and low quality habitat (coefficient: 2.319) were both positively associated with onsite *p* at EHW (*P* < 0.05). This is consistent with expectations of faster invasion in regions of poor habitat. However, the same result was not observed at PP, with none of the habitat variables or habitat quality demonstrating a significant relationship with *p*.

For the GLMMs modelling offsite variables, we found the only reliable predictor of *p* to be distance from the release zone. Distance predicted *p* robustly (*P* < 0.01) for all treatments, which were estimated to decrease by 0.6% to 1.2% per metre from the release zone (S8 Table). These estimates remained relatively consistent over time as the invasion progressed outward. Distance was not predictive of trap yields. For all seasons and sites, we observed no significant relationship between habitat and trap yields, save for in W2 at EHW wherein local abundance of window screens was positively linked with trap yield (*P* < 0.05). Offsite *p* was generally independent of housing covariates and habitat quality (S8 Table). The exceptions at EHW were screening in W2 and low quality habitat at D1 and W2, all of which correlated negatively with *p*. At PP, building height was predictive of *p* at W1 and D2, wherein local abundance of low set buildings was associated with lower *p*. None of these variables performed robustly across seasons or sites.

**References**

[1] ESRI. Arc GIS 10.2.2. Environmental Systems Research Institute; 2014.

[2] Barton N, Turelli M. Spatial waves of advance with bistable dynamics: cytoplasmic and genetic analogues of Allee effects. Am Nat. 2011;178: E48-75.

[3] Barrera, R. Spatial stability of adult *Aedes aegypti* populations. Am J Trop Med Hyg. 2011;85: 1087-92.

[4] Williams CR, Long SA, Webb CE, Bitzhenner M, Geier M, Russell RC, et al. *Aedes* *aegypti* population sampling using BG-Sentinel traps in North Queensland Australia: statistical considerations for trap deployment and sampling strategy. J Med Entomol 2007;44: 345-50.

[5] Hoffmann AA, Goundar AA, Long SA, Johnson PH, Ritchie SA. Invasion of Wolbachia at the residential block level is associated with local abundance of *Stegomyia aegypti*, yellow fever mosquito, populations and property attributes. Med Vet Entomol. 2014b;28: 90-7.

[6] Rousset F. spaMM: Mixed models, particularly spatial GLMMs. R package version 1.1. 2014.

[7] Matérn B. Spatial variation. Stockholm: State Forestry Research Institute. 1960.
